# Experimental evidence that apologies promote forgiveness by communicating relationship value

**DOI:** 10.1038/s41598-021-92373-y

**Published:** 2021-06-23

**Authors:** Daniel E. Forster, Joseph Billingsley, Jeni L. Burnette, Debra Lieberman, Yohsuke Ohtsubo, Michael E. McCullough

**Affiliations:** 1 U.S. Combat Capabilities Development Command Army Research Laboratory, Aberdeen Proving Ground, MD USA; 2 University of Miami, Coral Gables, FL USA; 3 North Carolina State University, Raleigh, NC USA; 4 Kobe University, Kobe, Japan; 5 University of Tokyo, Tokyo, Japan; 6 University of California, San Diego, CA USA

**Keywords:** Human behaviour, Social evolution

## Abstract

Robust evidence supports the importance of apologies for promoting forgiveness. Yet less is known about how apologies exert their effects. Here, we focus on their potential to promote forgiveness by way of increasing perceptions of relationship value. We used a method for directly testing these causal claims by manipulating both the independent variable and the proposed mediator. Namely, we use a 2 (Apology: yes vs. no) × 2 (Value: high vs. low) concurrent double-randomization design to test whether apologies cause forgiveness by affecting the same causal pathway as relationship value. In addition to supporting this causal claim, we also find that apologies had weaker effects on forgiveness when received from high-value transgressors, suggesting that the forgiveness-relevant information provided by apologies is redundant with relationship value. Taken together, these findings from a rigorous methodological paradigm help us parse out how apologies promote relationship repair.

## Introduction

Humans enact an extensive set of strategies to seek forgiveness after they have harmed others^1^. Verbal apologies and offers of compensation are among the most common and effective of these strategies^2^, changing victims’ attitudes toward their transgressors in many ways (for example, by increasing trust and feelings of friendship, and by reducing anger and the desire for revenge^3,4^). But how do apologies produce these changes?

To gain purchase on this question, we adopt an adaptationist perspective that situates forgiveness in the context of selection pressures stemming from life in complex, enduring social environments^5^. Humans form and maintain relationships with others in order to obtain resources essential for fitness, but doing so necessarily exposes them to potential harm, betrayal, and exploitation. Fraught with both opportunity and peril, this complex social landscape imposes selection for psychological mechanisms that assemble and manage the individual’s social network in a way that maximizes fitness benefits while minimizing costs. If they are to accomplish this task efficiently and effectively, such mechanisms should attend carefully to cues in the social environment that reliably predict the net fitness gains (or losses) that the individual stands to reap from future interactions with specific associates^5–8^. Such cues likely include kinship, shared interests, ingroup status, and a history of successful cooperation. For each associate, the psychological mechanisms tasked with managing the individual’s social network should integrate these cues into a summary cognitive representation that adaptationist researchers term “relationship value”—an internal index that captures the extent to which another person possesses attributes likely to raise the welfare of the person who holds that representation^7^. These perceptions affect whether people decide to pursue or forgo specific relationship opportunities across a range of contexts, giving rise to society-level “biological markets” characterized by partner choice^9,10^.

Adaptationist researchers posit that relationship value takes on acute importance in the wake of interpersonal harm^1,11–13^—a time when, for victims, the fitness stakes are especially high but the optimal response may be unclear. Avoiding the transgressor carries the advantage of eliminating the prospect of future harm, but carries the cost of potentially alienating oneself from closed, tight-knit groups in a shared social network. Vengeance may also reduce future harms, by deterring the transgressor (and perhaps others) from inflicting costs; but if deterrence fails, revenge might set in motion cycles of escalating counter-retaliation. Beyond these drawbacks, both avoidance and revenge suffer the same potentially severe shortcoming, namely that they jeopardize what may ultimately be a large stream of benefits from future cooperation with the transgressor, if the relationship could be sustained on favorable enough terms. To choose adaptively from among such consequential behavioral options requires above all else an estimate of the fitness gains that might accrue from ongoing interaction with the transgressor—which is why we, in keeping with prior adaptationist approaches, regard relationship value as a fundamental feature of any cognitive system that natural selection might design for the function of implementing forgiveness^3,6,14^. On this view, perceptions of the transgressor’s relationship value are a key factor determining whether victims’ interpersonal motivations toward a transgressor incline toward vengeance and avoidance, or toward benevolence, and thus whether damaged relationships are ultimately more likely to be repaired or left unrestored.

Given the crucial role that relationship value should play in the decision-making systems that regulate interpersonal motivation and behavior, particularly in the wake of harm, researchers postulate that changes in such perceptions provide a major mechanism through which apologies and other conciliatory gestures reliably promote forgiveness^3,4,14^. From this perspective, an act of harm or betrayal tends to reduce the victim’s valuation of the transgressor, and their motivation toward the transgressor accordingly turns more vengeful, more avoidant, and less benevolent. A transgressor who is averse to the consequences of these motivational shifts then confronts the question of how best to undo them. According to the adaptationist model, apologies, compensation offers, and other conciliatory gestures are effective tactics for doing so because they serve in part to increase victims’ perceptions of the transgressor’s relationship value: apologies upregulate the very representation that suffered in the wake of the transgression. Upon this view, then, victims tend to forgive their apologetic transgressors, and in turn prefer to continue interacting with transgressors vs. with alternative partners moving forward, because transgressors’ apologies directly target victims’ dynamically fluctuating assessments of the transgressor’s relationship value.

The claim that apologies foster forgiveness via changes in perceptions of relationship value rests on the idea that apologies work via causal mediation: apologies increase victims’ perceptions of the transgressor’s relationship value, which in turn increase forgiveness^3,5,6,14^. Researchers commonly test such mediational hypotheses with observational data from cross-sectional or longitudinal studies, but non-experimental data rarely license us to make definitive cause-and-effect inferences^15^. To study mediation with greater rigor, researchers often conduct hybrid experimental-observational studies that enable them to assess whether a hypothesized cause (e.g., apology) changes scores on both the hypothesized mediator (e.g., perceived relationship value) and the outcome (e.g., forgiveness). Having done so, they then evaluate the mediational hypothesis with statistical tests that focus on the observed correlation of the mediator with the outcome. Although such hybrid designs offer better evidence for mediation than purely non-experimental data, even hybrid designs leave alternative interpretations open because they cannot determine whether the hypothesized mediator causes the outcome, whether the outcome causes the mediator, or whether the mediator and outcome share a common cause^15^.

To obtain direct causal evidence for the entire process model, researchers may also include a manipulation that produces different levels of the mediator (M*, where M is measured and M* is manipulated) to assess whether the mediator truly causes changes in the outcome^16,17^. A “concurrent double-randomization”^17^ experiment, in which levels of the predictor (X) and mediator (M*) are concurrently manipulated, can take this approach further by providing direct causal evidence that (1) the predictor causes changes in the outcome (Y–X); (2) the mediator causes changes in the outcome (Y–M*); and (3) the predictor causes changes in the outcome by influencing the mediator (i.e., effect of Y–X depends on M*). By manipulating *and* measuring the mediator (M* and M, respectively), one could assess mediation using both standard tests of indirect effects (Y–X via M) and experimental tests of moderation (Y–X + M* + XM*). In many areas of research, it is difficult to experimentally manipulate mediators, but in the case of research on forgiveness, existing techniques for manipulating proxies for relationship value (such as interpersonal commitment and closeness^18^) provide a potential way forward.

For example^19^, found that participants reported more forgiveness for a specific relationship partner when participants had been experimentally induced to focus on their commitment to the partner instead of their independence from the partner. In a separate effort^20^, found that participants reported more inclination to forgive after they had been subliminally primed with the name of a close relationship partner rather than with the name of a non-close partner. Most recently^6^, found that people reported being more forgiving when imagining a transgression from high-value, versus low-value, relationship partners. If used in tandem with experimental manipulations of apology, such techniques hold promise for creating experiments that might yield valid inferences about mediation.

However, limitations in^6,19,20^ invite some circumspection about the utility of their particular techniques for manipulating perceived relationship value. In both sets of experiments, researchers studied participants’ responses to hypothetical scenarios, which may not correspond well to people’s real-life responses to betrayals^21,22^. Also^20^, relied on relatively subtle social priming methods to manipulate relationship closeness—methods that have drawn some scrutiny on both empirical and theoretical grounds^23,24^. Additionally, a registered replication effort involving sixteen independent labs failed to reproduce the effect of commitment on self-reported forgiveness responses to a hypothetical betrayal^25^.

Despite the potential limitations of these previous efforts, the causal effect of relationship value on forgiveness remains plausible and testable. We advocate the use of a method that induces greater relationship value among people in real-time interactions: the Relationship Closeness Induction Task (RCIT^26^). In the RCIT, participants share intimate personal information in a brief and natural conversation, which causes people to value each other much more than they value strangers with whom they have no interaction history. If relationship value is a causal mediator in the apology-forgiveness effect, and the closeness which the RCIT induces is a valid proxy for relationship value^18^, then the RCIT should serve as an excellent tool for evaluating whether the effects of apology on forgiveness are due to their intermediate effects on perceived relationship value.

### Testing mediation with moderation

Framing a question about the joint effects of apologies and perceived relationship value on forgiveness as a test of moderation is particularly interesting because of the placement of perceived relationship value and apology in the natural history of an interpersonal transgression. Pre-transgression relationship value, existing as it does before any transgression can occur, creates a context that may influence how victims perceive transgressions and subsequent transgressor behaviors. For instance, relationship value could promote forgiveness either by making a transgression seem less outrageous from the outset or by making the transgressor more forgivable after the fact^3,5,11,27^. Alternatively, and perhaps particularly in the case of severe transgressions among close relationship partners, relationship value might make the transgression seem *more* outrageous because victims may see it as an especially egregious violation of established relationship norms (though some observational evidence runs counter to this latter suggestion: marital infidelity, for example, is less likely to lead to divorce in long marriages than in short ones^28^). Apologies, in contrast, can influence forgiveness only *after* the transgression has taken place. Victims’ perceptions of transgressors’ relationship value could therefore exert their apology-modifying effects by either *facilitating* or *inhibiting* the effect of apologies on forgiveness.

**Figure 1:**
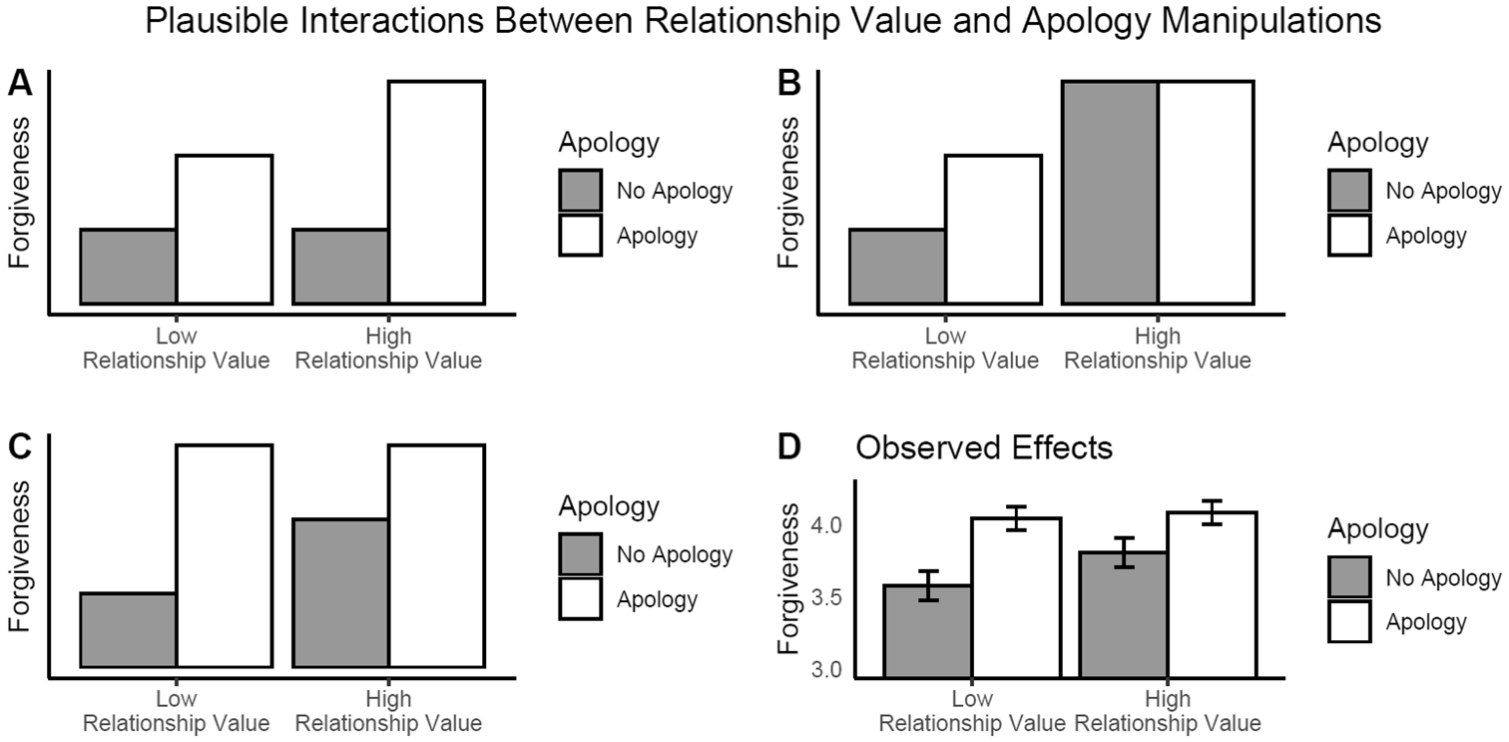
Three plausible interactions between levels of relationship value and apology manipulations (**A**–**C**), as well as the observed interaction (**D**). In (**A**), the effects of the relationship value and apology manipulations are greater than the sum of their isolated effects. In (**B**), manipulation of relationship value provides the strongest forgiveness-relevant information, rendering the apologies ineffective in the high relationship value condition. In (**C**), apologies provide the strongest forgiveness-relevant information, rendering the relationship value manipulation ineffective in the apology condition. To produce the figure in (**D**), we computed a simple composite score from the TRIM-NCO along with error bars representing 95% confidence intervals; the pattern we observe in our data is the most similar to (**C**).

Figure 1 illustrates three plausible alternatives for how relationship value might moderate the effects of apologies on forgiveness. First, as in Fig. 1A, pre-transgression relationship value might *enhance* the effect of an apology—by making it seem more sincere, for instance^29^. On this view, relationship value amplifies the effects of an apology *pragmatically* by creating a relational context that changes its meaning. Second, as in Fig. 1B, pre-transgression relationship value might contain information about the desirability of forgiving that overshadows the (presumably) lower-quality information provided by an apology, with the result that the effects of apologies have smaller effects in relationships in which victims perceive their transgressors to possess high relationship value. We also entertain a third possibility, illustrated in Fig. 1C: if, rather than apology, it is pre-transgression relationship value that provides the lower-quality information about how a partner might respond after a transgression, an apology could conceivably carry so much surplus informational value as to overshadow the effect of pre-transgression relationship value, with the result that perceived relationship value prior to the transgression has smaller effects on forgiveness in the presence of an apology than in the absence of an apology. Whether pre-transgression relationship value facilitates or inhibits the efficacy of an apology on forgiveness, the nature of the interactive effects will inform the nature of the mediational role of relationship value. Finally, we note that other interaction patterns are technically possible, but we restricted the examples presented here to those that depict both apologies and relationship value as having a causal effect on forgiveness, as is assumed by our model and supported by previous findings^3–6,14,30^.

### The current experiment

Drawing upon our understanding of psychological closeness as an index of perceived relationship value^5,18,26^, in tandem with our theoretical understanding of how apologies and relationship value influence forgiveness^3–6,14^, we sought to test whether apologies cause forgiveness through their intermediate influence on victims’ subjective valuations of their transgressors. To do so, we deployed a 2 (Transgressor Value: High vs. Low) × 2 (Apology: Yes vs. No) between-subjects design in the context of a manufactured transgression based on a common essay-writing paradigm^31,32^. Specifically, we first used the RCIT^26^ to influence participants’ perceptions of a novel interaction partner’s social value. We then introduced participants to a group-based essay writing task, in the course of which participants received insulting feedback on their essay from a fellow group member (whom we term “the transgressor”). In this context, we manipulated whether or not the transgressor is the group member with whom the participant completed the RCIT (Transgressor Value: High vs. Low), as well as whether or not the transgressor apologized (Apology: Yes vs. No). At various stages in the experiment, we also measured participants’ perceptions of the transgressor’s relationship value. Following the apology manipulation, we also measured participants’ willingness to forgive the transgressor. Finally, we measured participants’ behavioral preferences for interacting with a transgressor versus with a neutral third party, allowing us to examine whether apologies also influence partner choice via their intermediate effects on relationship value.

This concurrent double-randomization design^17^ affords a number of advantages. First, double randomization shields both independent variables from the potential confounding influences of personality and individual difference variables that have been found to correlate with forgiveness (e.g., victim’s agreeableness^33^ and just-world beliefs^34^). This enables us to test both the hypothesis that pre-transgression relationship value causes forgiveness, and the hypothesis that apologies cause forgiveness. Second, our experiment can be viewed as a *blockage* design^17^, which is a specific concurrent double-randomization approach where one level of the mediator (i.e., transgressor value) serves to eliminate or minimize—that is, block—the predictor’s (i.e., apology) influence on the outcome (i.e., forgiveness), while the other level of the mediator remains free to vary, thus allowing the predictor to exert its full influence on the outcome. Specifically, the “high” level of the mediator (i.e., increased relationship value via RCIT^26^ prior to the transgression) represents the specific level that serves to block the effects of the apology manipulation on forgiveness, assuming they both operate on the same underlying mechanisms. By contrast, the “low” level of the mediator (i.e., no interaction prior to the transgression) represents the level at which the mediator is free to vary—that is, we did not seek to experimentally reduce the effects of apologies on forgiveness by constraining participants to experience an artificially low level of the transgressor’s relationship value (e.g., by creating a negative interaction prior to the transgression). This blockage design allows us to directly test the specific hypothesis that relationship value influences *how* apologies cause forgiveness (i.e., whether apologies affect the same causal pathway as pre-transgression relationship value^16,17^). Third, by *measuring* (rather than merely manipulating) perceptions of relationship value, we are able to use traditional mediation analysis of indirect effects, as well as moderation analyses of our experimental manipulations, to test the hypothesis that apologies cause forgiveness through their intermediate influence on relationship value.

## Results

**Figure 2:**
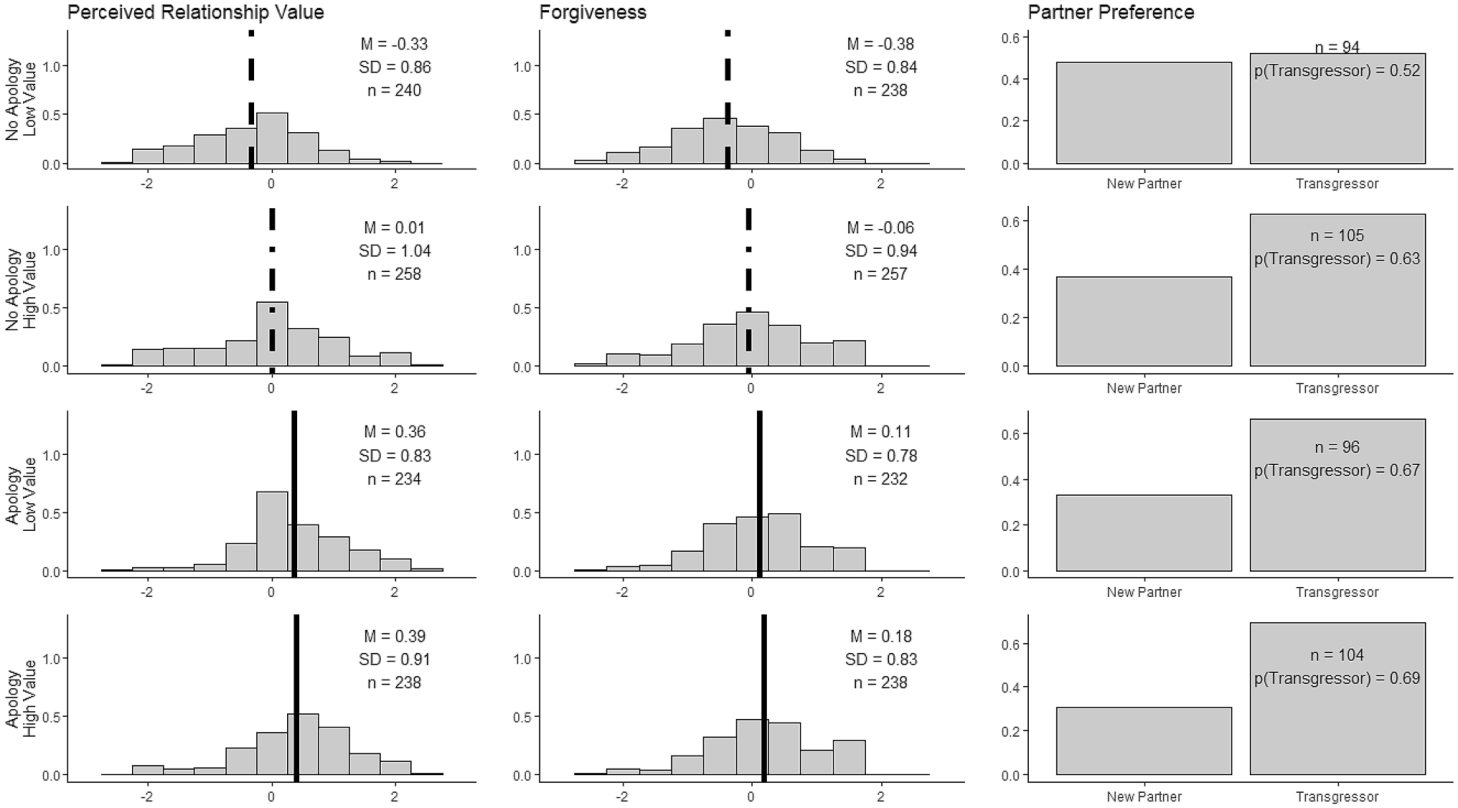
Histograms of participants’ factor scores representing their perceptions of the transgressor’s relationship value (post-apology), their forgiveness toward the transgressor, and bar charts of the proportion of participants who preferred to interact with their transgressors, across all four conditions. Vertical lines in histograms represent the mean. Means and standard deviations are displayed for continuous outcomes, proportion who chose interact with their transgressors are displayed for Partner Preference, and number of observations are displayed for all outcomes. For continuous outcomes, line types (solid, dashed, dot-dashed) which appear the same are not significantly different from each other.

Descriptive analyses and simple t-tests were conducted in R 3.5.1^35^. All structural equation models were conducted using Mplus version 7^36^. Methods and predictions were preregistered on the Open Science Framework (https://osf.io/x3qv9/?view_only=d3949f18fad74fc9b931c1726500b483). See Fig. 2 for histograms and descriptive statistics of our primary variables across each condition. Although our hypotheses and predictions were directional, all models were evaluated with two-sided null hypothesis significance tests.

### Manipulation check. Did the relationship value manipulation cause increased liking, closeness, similarity, and desire to befriend?

To replicate the analyses of^37^ and further validate the RCIT as a method of closeness induction, we examined mean differences between participants’ feelings toward the participant with whom they completed the RCIT versus their feelings toward two other group members with whom participants did not engage in the RCIT, using paired-sample t-tests on each of the four manipulation check items separately (see Table 1). Because participants completed these items with respect to each of three other group members, we averaged across the two non-RCIT targets for each item.

**Table 1:** Means, standard deviations, and effect sizes of the relationship closeness induction task for different manipulation check items.

	Close	Friends	Like	Similar
	M (SD)	M (SD)	M (SD)	M (SD)
RCIT partner	5.40 (2.02)	6.57 (1.88)	6.94 (1.65)	5.66 (2.02)
Non-RCIT partners	2.55 (1.81)	4.11 (1.76)	4.19 (1.67)	3.25 (1.88)
*Hedges’ g*	*1.30*	*1.20*	*1.36*	*1.01*

Hedge’s *g* represents the difference between participants’ responses for RCIT partners and non-RCIT partners in standard deviation units.

Similarly to^26^, we found that participants: (1) felt closer to their RCIT partners than to the other two group members, t(970) = 40.459, *p* < 0.001, Hedges’ *g* = 1.30; (2) expressed a greater desire to befriend the RCIT partners, t(970) = 37.38, *p* < 0.001, Hedges’ *g* = 1.20; (3) liked their RCIT partners more, t(970) = 42.33, *p* < 0.001, Hedges’ *g* = 1.36; and (4) felt more similar to their RCIT partners, t(970) = 31.52, *p* < 0.001, Hedges’ *g* = 1.01. Unsurprisingly, participants rated their RCIT partners more highly on the composite of all four items (M = 6.14; SD = 1.62) than they rated non-RCIT group members (M = 3.52; SD = 1.51), t(970) = 45.58, p < 0.001, Hedges’ *g* = 1.67.

### The complete model: predictions 1–3

**Figure 3:**
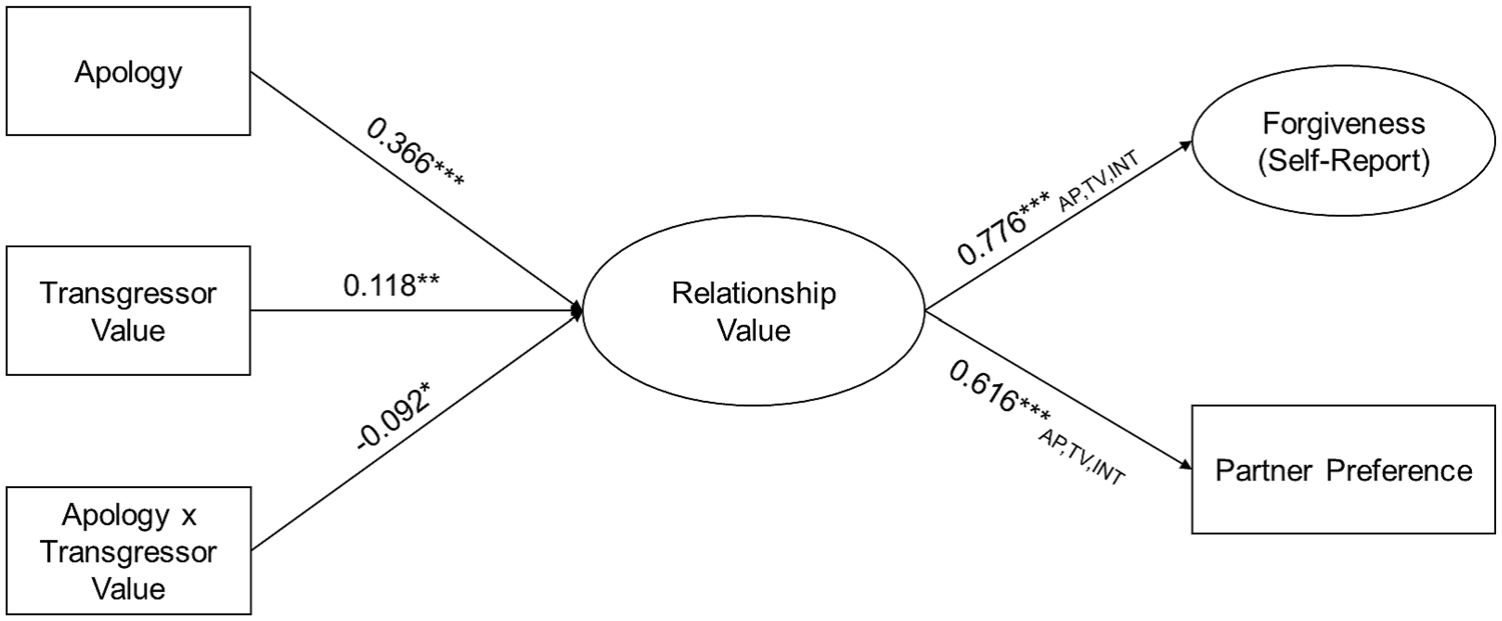
Path model depicting unstandardized direct and indirect effects of the apology (− 1 = no apology, 1 = apology) and transgressor value (− 1 = Low, 1 = High) manipulations, as well as their interaction, on measured relationship value, forgiveness, and preference to interact with the transgressor. A complete list of direct and indirect effects is in Table 2. Significant indirect effects are marked by subscripts from the mediator to the outcome: *AP* apology, *TV* transgressor value, *INT* interaction. ***p < 0.001, **p < 0.01 *p < 0.05.

**Figure 4:**
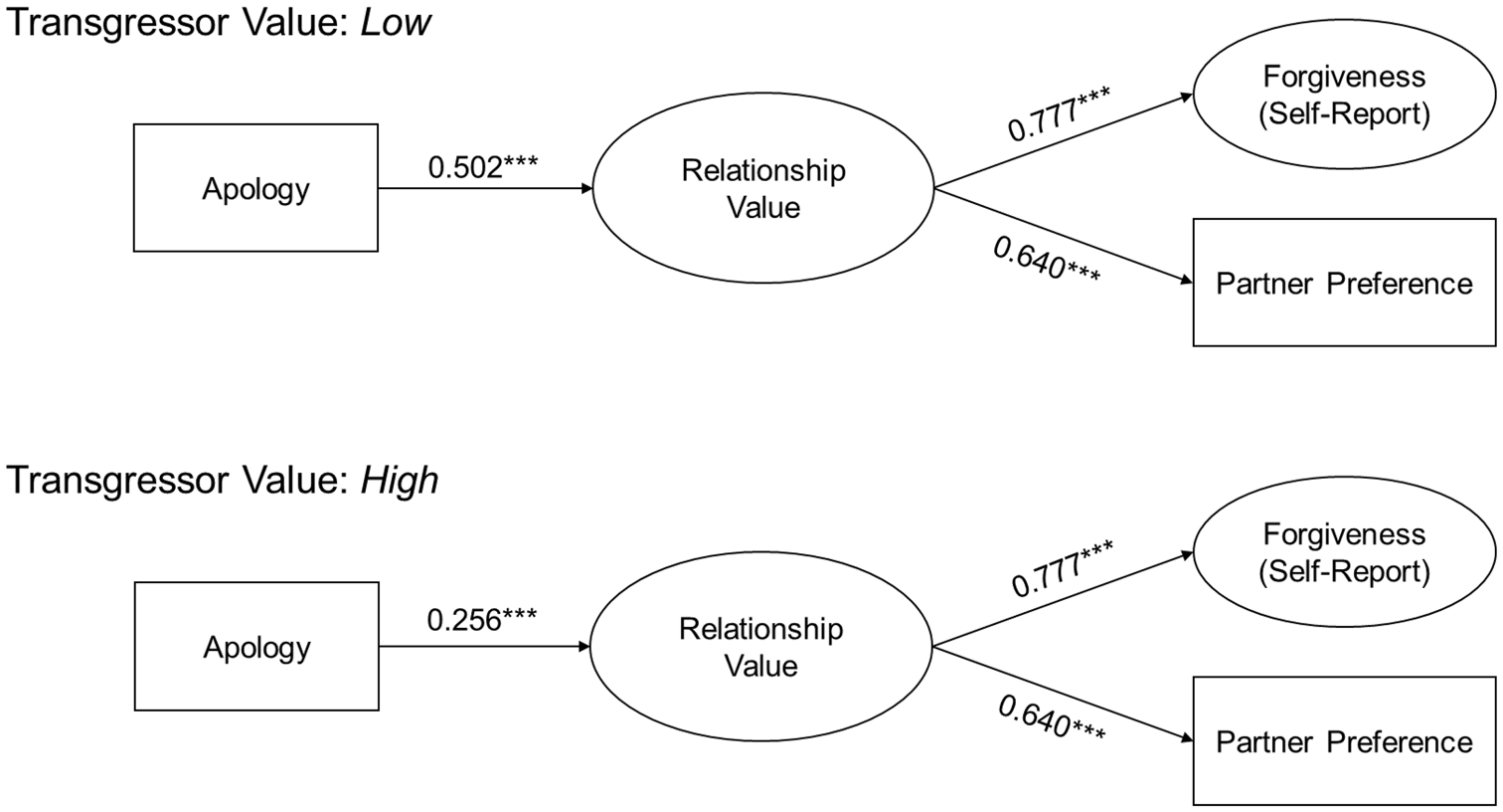
Path model depicting unstandardized simple effects of apologies across levels of transgressor value. A complete list of direct and indirect simple effects is in Table 3. Significant differences in effects across levels of relationship value are conveyed by the interaction terms reported in Fig. 3 and Table 2. ***p < 0.001.

Although we preregistered that we would test our predictions with separate models, we realized after the fact that we could more efficiently test all of our hypotheses in a single structural equation model (see Fig. 3). In this model, we effect-coded whether participants were insulted by their partner from the RCIT (− 1 = low transgressor value, 1 = high transgressor value) and whether they received an apology (− 1 = no apology, 1 = apology). We also computed the interaction from these effect-coded variables, which allowed us to interpret the effect of each variable as a true main effect, rather than as the simple effects we would have obtained with dummy coding. Therefore, the magnitude of our manipulated variables should be interpreted as comparisons between the average across all participants (i.e., when all manipulated variables are 0) and the participants assigned to the condition with a value of 1 on the manipulated variables. We estimated standard errors in the final model using 1000 bootstrap samples. The final model fit the data well, χ^2^(292) = 1131.374, *p* < 0.001, CFI = 0.988, RMSEA = 0.054, 90% CI [0.051, 0.058]. Path coefficients for this model are summarized in Table 2. To probe interaction effects, we also conducted a grouping analysis to test the effects of apologies across the levels of the transgressor’s value (Table 3, Fig. 4). We provide detailed predictions below.

**Table 2:** Path coefficients for the direct and indirect effects in the main model.

Regression	Estimate (SE)	z	*p*	95% CI
**RV~**				
Apology	**0.366 (0.038)**	**9.749**	**< 0.001**	**[0.293, 0.440]**
Value	**0.118 (0.036)**	**3.332**	**0.001**	**[0.049, 0.188]**
Apology × value	**− 0.092 (0.036)**	**− 2.538**	**0.011**	**[− 0.164, − 0.021]**
**Forgiveness~**				
RV	**0.776 (0.018)**	**42.596**	**< 0.001**	**[0.741, 0.812]**
Apology	**− 0.087 (0.023)**	**− 3.703**	**< 0.001**	**[− 0.133, − 0.041]**
Via RV	*0.285 (0.029)*	*9.79*	< *0.001*	*[0.228, 0.342]*
Total	*0.198 (0.032)*	*6.199*	< *0.001*	*[0.135, 0.260]*
Value	**0.017 (0.022)**	**0.747**	**0.455**	**[− 0.027, 0.061]**
Via RV	*0.092 (0.028)*	*3.336*	< *0.001*	*[0.038, 0.146]*
Total	*0.109 (0.032)*	*3.405*	< *0.001*	*[0.046, 0.171]*
Apology × Value	**0.002 (0.023)**	**0.095**	**0.924**	**[− 0.042, 0.046]**
Via RV	*− 0.072 (0.028)*	*− 2.242*	*0.03*	*[− 0.127, − 0.016]*
Total	*− 0.070 (0.031)*	*− 2.242*	*0.01*	*[− 0.130, − 0.009]*
**Partner Pref.~**				
RV	**0.616 (0.049)**	**12.489**	**< 0.001**	**[0.520, 0.713]**
Apology	**− 0.088 (0.066)**	**− 1.328**	**0.184**	**[− 0.218, 0.042]**
Via RV	*0.226 (0.028)*	*7.936*	< *0.001*	*[0.170, 0.282]*
Total	*0.138 (0.066)*	*2.079*	*0.04*	*[0.008, 0.268]*
Value	**0.014 (0.064)**	**0.214**	**0.831**	**[− 0.112, 0.139]**
Via RV	*0.073 (0.022)*	*3.29*	< *0.001*	*[0.029, 0.116]*
Total	*0.087 (0.066)*	*1.31*	*0.19*	*[− 0.043, 0.216]*
Apology × value	**0.006 (0.063)**	**0.098**	**0.922**	**[− 0.155, 0.129]**
Via RV	*− 0.057 (0.023)*	*− 2.484*	*0.01*	*[− 0.102, − 0.012]*
Total	*− 0.051 (0.066)*	*− 0.774*	*0.44*	*[− 0.179, 0.078]*

Coefficients are unstandardized, with coefficients pertaining to partner preference reported in log-odds. Confidence intervals were estimated using 1000 bootstrap samples. To distinguish direct effects from indirect and total effects, model direct effects are in bold while model indirect and total effects are italicized.

**Table 3:** Path coefficients for the simple effects of the apology manipulation across levels of the transgressor value manipulation.

Regression	Estimate (SE)	z	*p*	95% CI
**Low-value transgressor**				
RV~				
Apology	**0.502 (0.056)**	**9.018**	**< 0.001**	**[0.393, 0.610]**
Forgiveness~				
RV (c1)	**0.777 (0.028)**	**27.774**	**< 0.001**	**[0.722, 0.832]**
Apology	**− 0.113 (0.036)**	**− 3.107**	**0.002**	**[− 0.185, − 0.042]**
Via RV	*0.390 (0.045)*	*8.674*	< *0.001*	*[0.302, 0.478]*
Total	*0.277 (0.045)*	*6.200*	< *0.001*	*[0.189, 0.364]*
Partner Pref. ~				
RV (c2)	**0.640 (0.048)**	**13.458**	**< 0.001**	**[0.547, 0.734]**
Apology	**− 0.133 (.092)**	**− 1.440**	**0.150**	**[− 0.313, 0.048]**
Via RV	*0.321 (0.042)*	*7.566*	< *0.001*	*[0.238, 0.404]*
Total	*0.189 (0.090)*	*2.085*	*0.037*	*[0.011, 0.366]*
**High-value transgressor**				
RV~				
Apology	**0.256 (0.050)**	**5.137**	**< 0.001**	**[0.158, 0.353]**
Forgiveness~				
RV (c1)	**0.777 (0.028)**	**27.774**	**< 0.001**	**[0.722, 0.832]**
Apology	**− 0.076 (0.032)**	**− 2.399**	**0.016**	**[− 0.138, − 0.014]**
Via RV	*0.199 (0.039)*	*5.047*	< *0.001*	*[0.122, 0.276]*
Total	*0.123 (0.045)*	*2.738*	*0.006*	*[0.035, 0.211]*
Partner Pref.~				
RV (c2)	**0.640 (0.048)**	**13.458**	**< 0.001**	**[0.547, 0.734]**
Apology	**− 0.097 (0.092)**	**− 1.056**	**0.291**	**[− 0.254, 0.101]**
Via RV	*0.164 (0.034)*	*4.845*	< *0.001*	*[0.097, 0.230]*
Total	*0.087 (0.095)*	*0.922*	*0.357*	*[− 0.098, 0.273]*

Coefficients are unstandardized, with coefficients pertaining to partner preference reported in log-odds. Confidence intervals were estimated using 1000 bootstrap samples. To distinguish direct effects from indirect and total effects, model direct effects are in bold while model indirect and total effects are italicized. Paths from perceived relationship value (RV) to self-report Forgiveness (c1) and Partner Preference (c2) were constrained to be equal across levels of transgressor value.

#### Prediction 1

We predicted that participants would assign higher relationship value to transgressors with whom they completed the RCIT (1a), and to transgressors who apologized (1b). Although we did not preregister a hypothesized direction of the interaction, as there is no principled a priori reason to expect pre-transgression relationship value to either amplify or inhibit the effects of apologies, our reading of^16^ and^17^ led us to understand that if apologies work by increasing the transgressor’s perceived relationship value, then we might expect a significant interaction between the relationship value and apology manipulations (1c).

#### Prediction 2

We predicted participants would be more forgiving of transgressors with whom they completed the RCIT (2a) and from whom they received an apology (2b). As in 1c, we did not have any predictions regarding the nature of the interaction of transgressor value and apology, but still tested the interaction to inform us about the nature of relationship value in the apology-forgiveness link (2c). We also predicted that participants’ perceptions of the transgressors’ relationship value would appear (via the correlation of measured relationship value and forgiveness) to mediate these effects (2d).

#### Prediction 3

With respect to the partner choice dependent variable, we made predictions (3a–3d) that were parallel to those we made with respect to the forgiveness dependent variable (Predictions 2a–2d).

### Prediction 1. Did manipulated transgressor value and apology change participants’ perceptions of the transgressor’s relationship value?

#### Prediction 1a

Compared to participants on average, participants in the high-value condition assigned more relationship value to the transgressor, b = 0.118, se = 0.036, *p* = 0.001, 95% CI [0.049, 0.188].

#### Prediction 1b

Compared to participants on average, participants who received an apology assigned more relationship value to the transgressor, b = 0.366, se = 0.038, *p* < 0.001, 95% CI [0.293, 0.440].

#### Prediction 1c

The effect of apology on perceived relationship value was lower for participants in the high-value condition than for participants on average, as evinced by a negative interaction, b = -0.111, se = 0.037, *p* = 0.003, 95% CI [-0.164, -0.021]. In an analysis grouped by transgressor value, apologies increased relationship value more in the low-value transgressor condition, b = 0.502, se = 0.056, *p* < 0.001, 95% CI [0.393, 0.610], than in the high-value transgressor condition, b = 0.256, se = 0.050, *p* < 0.001, 95% CI [0.158, 0.353].

### Prediction 2. Did manipulated transgressor value and apology promote forgiveness through their intermediate effects on perceived relationship value?

#### Prediction 2a

Compared to participants on average, participants in the high-value transgressor condition forgave more, b = 0.109, se = 0.032, *p* = 0.001, 95% CI [0.046, 0.171].

#### Prediction 2b

Compared to participants on average, participants who received an apology forgave more, b = 0.198, se = 0.032, *p* < 0.001, 95% CI [0.135, 0.260].

#### Prediction 2c

Consistent with the hypothesis that apology influences forgiveness via its intermediate influence on perceived relationship value, the effect of apology on forgiveness was lower for participants in the high-value transgressor condition than for participants on average, as evinced by the significant negative interaction of apology and transgressor value, b = -0.070, se = 0.031, *p* = 0.025, 95% CI [-0.130, -0.009]. In an analysis grouped by transgressor value condition, apologies increased forgiveness more in the low-value transgressor condition, b = 0.277, se = 0.045, *p* < 0.001, 95% CI [0.189, 0.364], than in the high-value transgressor condition, b = 0.123, se = 0.045, *p* = 0.006, 95% CI [0.035, 0.211].

#### Prediction 2d

Also consistent with the idea that apology influences forgiveness via its intermediate effect on perceived relationship value, participants’ measured perceptions of the transgressor’s relationship value appeared, via their correlation with forgiveness, to mediate the effect of apology on forgiveness, b = 0.285, se = 0.029, *p* < 0.001, 95% CI [0.228, 0.342]. Similarly, participants’ measured perceptions of the transgressor’s relationship value appeared to mediate the effect of the transgressor value manipulation on forgiveness, b = 0.092, se = 0.028, *p* = 0.001, 95% CI [0.038, 0.146].

In addition, the effect of apology on forgiveness was less strongly mediated by perceived relationship value among participants with high-value transgressors than among participants with low-value transgressors, as confirmed by the significant interaction term, b = -0.072, se = 0.028, *p* = 0.011, 95% CI [-0.127, -0.016]. In an analysis grouped by transgressor value, the indirect effect of apologies upon forgiveness was stronger among participants in the low-value transgressor condition, b = 0.390, se = 0.045, *p* < 0.001, 95% CI [0.302, 0.478], than in the high-value transgressor condition, b = 0.199, se = 0.039, *p* < 0.001, 95% CI [0.122, 0.276]. This result is consistent with the idea that the attenuated effect of apologies on forgiveness in high-value relationships is partially due to the attenuated effect of apologies on measured relationship value in high-value relationships.

### Prediction 3. Did participants prefer to interact with transgressors who engaged in the RCIT and/or who apologized? Were these effects mediated by perceived relationship value?

For these results, we reported confidence intervals for odds ratios (OR), which represent the likelihood of participants choosing to continue interacting with the transgressor, instead of with a new partner with whom they had very little interaction (i.e., the group member who was neither an RCIT partner nor a transgressor). We also acknowledge that this outcome was only recorded for a subset of the sample (41%; N = 399), resulting in substantially less power to detect effects.

#### Prediction 3a

Compared to participants on average, participants in the high-value transgressor condition were no more likely to prefer continuing to interact with the transgressor than were participants in the low-value transgressor condition, b = 0.087, se = 0.066, *p* = 0.190, OR = 1.091, 95% CI [0.958, 1.241].

#### Prediction 3b

Compared to participants on average, participants who received an apology preferred to continue interacting with their transgressors, b = 0.138, se = 0.066, *p* = 0.038, OR = 1.148, 95% CI [1.008, 1.307].

#### Prediction 3c

The total main effects of the relationship value and apology manipulations on partner preference were not qualified by a significant interaction, b = -0.051, se = 0.066, *p* = 0.439, OR = 0.950, 95% CI [0.836, 1.081]. These results fail to support the hypothesis that apologies influence partner choice by making transgressors appear more valuable as relationship partners; however, our subsequent analysis offers a different approach to addressing this very question.

#### Prediction 3d

Participants’ perceptions of the transgressor’s relationship value appeared, via their correlation with participants’ partner choice decisions, to mediate the effect of apology on participants’ preference for continuing to interact with the transgressor, b = 0.226, se = 0.028, *p* < 0.001, OR = 1.798, 95% CI [1.250, 1.326]. Likewise, the effect of the relationship value manipulation on participants’ preference for continuing to interact with the transgressor was apparently attributable to its intermediate influence on participants’ measured perceptions of the transgressor’s relationship value, b = 0.073, se = 0.022, *p* = 0.001, OR = 1.076, 95% CI [1.029, 1.122]. Significant mediation thus occurred even though the relationship value manipulation did not have a significant total effect on partner choice, which should not be too surprising considering the greater statistical power for indirect than for total effects^38^. Additionally, the indirect effect of apology on partner preference was weaker among participants in the high-value transgressor condition than among participants in the low-value transgressor condition, as confirmed by the significant interaction term, b = -0.057, se = 0.023, *p* = 0.013, OR = 0.945, 95% CI [0.903, 0.988]. In an analysis grouped by transgressor value, the indirect effect of apologies upon partner preference was stronger among participants in the low-value transgressor condition, b = 0.321, se = 0.042, *p* < 0.001, OR = 1.379, 95% CI [1.269, 1.498], than in the high-value transgressor condition, b = 0.164, se = 0.034, *p* < 0.001, OR = 1.178, 95% CI [1.102, 1.259]. This result is consistent with the idea that the attenuated effect of apologies on preferences for interacting with the transgressor is partially due to the attenuated effect of apologies on measured relationship value in high-value relationships.

### Exploring why apologies were less effective for high-value partners: testing the resilience of perceived relationship value following the transgression

In our analyses above, relationship value was only considered following the apology manipulation. We also assessed perceptions of the transgressor’s relationship value following the transgression, but prior to the apology manipulation, enabling us to address whether the relationship value manipulation impacted immediate post-transgression perceptions of relationship value. In doing so, we find that the manipulation resulted in victims holding higher values for their transgressors if they had previously engaged in the closeness induction task, b = 0.223, se = 0.054, p < 0.001, 95% CI [0.118, 0.328]. Thus, the transgression did not bring victims’ perceptions of close transgressors to the same level as victims’ perceptions of non-close transgressors. These results suggest that apologies from close transgressors were less effective at increasing relationship value than apologies from non-close transgressors because substantial residual value was retained from the closeness manipulation despite the transgression.

## Discussion

Building upon theoretical and empirical developments in the evolutionary social sciences, researches have posited that the victims of interpersonal transgressions rely upon the value of maintaining a relationship with a transgressor in making decisions to forgive, and that apologies facilitate forgiveness by modifying victims’ perceptions of the transgressor’s relationship value^3–5,14^. Until now, however, these propositions have not received a rigorous test that could yield unambiguous evidence of causality.

With a concurrent double-randomization blockage design^17^ in which we sequentially manipulated perceived relationship value (with a task designed to help strangers feel closer to each other) and apologies, we obtained direct causal evidence for both of these propositions. The manipulation of relationship value promoted forgiveness, and apologies had weaker effects on forgiveness when received from high-value transgressors (Fig. 1D). These interference effects are direct evidence that apologies affect forgiveness through their intermediate effects on relationship value^17^.

### Implications of the current research

Our experimental evidence sheds light on two novel phenomena. First, we find that apologies are less effective in promoting forgiveness in nascent higher-value relationships *because* they are less effective in raising perceived relationship value in already higher-value relationships. Of course, our design could only be used to understand these effects in early stages of relationship formation; indeed, previous cross-sectional research has found that apologies are *more* effective among close relationship partners with a long history^29^. Therefore, we suspect that the relative impact of apologies and relationship value fluctuates over the course of the relationship, perhaps because over time the relationship provides information that moderates the effects of apologies, such as perceived sincerity^29,39^.

Second, we find that victims of high-value (and apologetic) transgressors exhibited a notable preference for interacting with transgressors even when given the opportunity to interact with someone who had never harmed them. We suggest that these findings are best understood in the context of reputation-based partner choice^9,10^. Specifically, we argue that forgiveness attitudes, existing on a continuum from hostility to friendliness^40^, represent how victims decide to allocate their social resources between retaining a relationship and developing new relationships. Our findings provide direct evidence that forgiveness functions to further develop an existing relationship. Future forgiveness research should take into consideration impacts from, and on, broader social networks^41–43^, to further understand the role of relationship value in driving forgiveness.

### Limitation and future directions

Here we discuss the primary constraints on generalizability that our study faces, and provide suggestions for how to extend generalizability in future research.

We begin with the sample, which in the case of our study was limited to strangers interacting for the first time. This limitation impairs our ability to draw conclusions about how victims respond to apologies from closer and longer-term relationship partners. Moreover, interactions in our paradigm took place online, raising intriguing questions about whether and how the online medium itself might shape the manner in which victims perceive and respond to both transgressions and apologies. Future studies on the buffering effects of relationship value, or the causal processes through which apologies work, could investigate these effects among coworkers, co-inhabitants, friends, romantic partners, and relatives. Although researchers could not experimentally induce these types of relationships, they could in principle create a group setting where members vary in pre-existing relationship value, then manipulate whether a close friend or family member commits a transgression. Ideally, such a paradigm could also be used to manipulate whether the context was online or face-to-face, so as to isolate possible influences of the medium on the causal chain. In addition, researchers could expand use of the RCIT paradigm—which involves only newly interacting strangers—by randomizing participants to interact either face-to-face, or online.

Our study also examined a limited range of transgressions and apologies, which of course can take numerous forms. McCullough et al.^27^, for instance, surveyed undergraduates who had recently experienced a transgression in their real lives, and found that while most participants experienced insults, many of them also experienced social rejection, partner neglect, infidelity, and relationship termination. Absent from McCullough et al.’s survey responses were other notable transgressions, such as property damage, theft, and bodily injury. To explore additional experimental techniques for inducing a transgression, researchers might look toward social exclusion paradigms (e.g., confederates excluding the focal participant from engaging in a group activity^44^); economic paradigms (e.g., confederates stealing monetary allocations^45^); or even methods for inducing physical pain in a laboratory setting (e.g., using the cold pressor test^46^). In so doing, they might also tailor the experimental methods they use for manipulating apologies so that they better match the transgression’s severity and transgressor’s perceived responsibility^47^. For instance, we designed our verbal apology and compensation offer to emulate transgressors’ strategies for seeking forgiveness following a *moderate* transgression^47^. Beyond matching apologies to transgression severity, researchers might extend work on how compensation offers and verbal apologies each uniquely influence forgiveness. Although the distinct effects of verbal apology and compensation feature prominently in some prior studies (e.g.,^48,49^), researchers can advance this work by examining how these unique effects influence the relationship value-forgiveness pathway.

Last, we acknowledge the limitations of our measurement tools for assessing the effects of transgressions and apologies on perceived relationship value and forgiveness. To measure relationship value, we relied upon a single self-report measure, to assess partner preference, we relied upon a single decision. In the case of relationship value, researchers could strengthen conclusions by conducting conceptual replications that use alternative self-report measures of relationship value—for example, measures of perceived goal instrumentality^12,13^ or welfare tradeoff ratios^50,51^. Additionally, future work should examine whether our observed effects could be detected using *behavioral* measures (e.g., verbal expressions of social value and forgiveness, or direct benefit-delivery in a subsequent interaction). As a final strategy for strengthening causal inference, researchers could measure, and thus explicitly account for, additional constructs that might serve as alternative mediators^17^—for example, perceived transgression severity^27,52^ and responsibility attributions^27,52,53^.

## Conclusions

We hope our results inspire a resurgence of interest in the idea that people’s perceptions of the quality of their relationships^19,20^, and of the value of their relationship partners^6^, play a central causal role in their decisions about whether, when, and why to forgive. Our results also provide reason for optimism that it might be possible in future work to obtain unambiguous causal evidence as to whether victims’ perceptions of their transgressors’ exploitation risk—which, along with perceived relationship value, is posited to be a key cognitive representation underlying people’s decisions about whether to forgive—really do exert a causal effect on forgiveness^3–5,14^. Although our findings provide clear evidence for causal mediation, we only scratched the surface of the broader causal network that characterizes interpersonal dynamics surrounding relationship building, conflict, repair, and dissolution. To build a more comprehensive model of how perceptions of relationship value impact forgiveness (and other interpersonal outcomes), social scientists would be wise to consider advances in causal analysis—with careful thought to study design and analysis, causal evidence of mediation could be reasonably built upon both experimental and cross-sectional data^54^. More generally, we hope these results renew hope among social psychologists, and close relationships researchers in particular, that unambiguous causal evidence for mediational hypotheses might be easier to obtain than we have come to expect.

## Methods

Before collecting data, we pre-registered all of our data collection and analysis procedures on the Open Science Framework (https://osf.io/x3qv9/?view_only=d3949f18fad74fc9b931c1726500b483). We note here any adjustments from our pre-registration plan. All procedures were approved by the University of Miami’s Institutional Review Board (protocol #20140553). All methods were carried out in accordance with relevant guidelines and regulations. Informed consent was obtained from all participants.

Participants were 971 workers from Amazon’s Mechanical Turk (Age: *M* = 35.22, SD = 10.92; 55.72% Female), all of whom consented to participate. Participants were assigned to one of four cells in a 2 (Transgressor Value: High vs. Low) × 2 (Apology: Yes vs. No) between-participants design. We guaranteed participants a minimum of $1.00 for participating in the experiment, with the promise of opportunities to earn additional money for completing the tasks within the experiment. In actuality, all participants who completed the experiment earned a $7.00 bonus, resulting in a total payment of $8.00 per participant. Participation was restricted to users from the United States with approval ratings at or above 90%.

Deviating from our pre-registration plan, we decided after the fact to test whether suspicious participants (n = 467) behaved differently from non-suspicious participants. Our goal in doing so was to determine whether the two groups could be combined inasmuch as the larger data set would provide more statistical power and avoid violating the rules of causal inference based on experimentation, as happens when participants are removed after random assignment^55^. Our analyses revealed that none of the path coefficients were significantly different between suspicious and non-suspicious participants (see “Supplemental materials” for full analysis); therefore, we retained all participants in the analyses reported. Still, we also conducted our analyses without suspicious participants, the results of which are available in the “Supplemental materials”.

### Procedure

We conducted the experiment using the Software Platform for Human Interaction Experiments (SoPHIE^56^), which enabled us to create authentic interactions between participants (see “Supplemental materials” for SoPHIE script). From their own computers, participants entered a virtual “waiting room” after consenting to participate. Once a second participant had entered the waiting room, the two participants were paired with each other. Participants were led to believe that there were actually three other participants within their group. In actuality, there was only the one other human participant, and interactions with even that participant were authentic only for the first part of the study. Participants were told that they would work on some of the study’s tasks as a four-person group, other tasks in pairs, and still other tasks independently.

### The relationship closeness induction task

We experimentally manipulated the transgressor’s value prior to the transgression by having participants participate in a modified version of the Relationship Closeness Induction Task (RCIT; Sedikides et al., 1999), with the other human participant from their four-person group. In the high-value condition, the RCIT partner would go on to insult the participant in a subsequent interaction (see below). In the low-value condition, another participant, rather than the RCIT partner, would deliver the subsequent insult. Stated differently, we used the RCIT to raise the perceived relationship value of either a subsequent transgressor or an innocent bystander.

In the RCIT, participants engaged in an open conversation with their partners, although their conversation was split into three timed sections (90 s, 3 min, and 5 min, respectively), with each section displaying topics for discussion. We modified some of the prompts on the RCIT to make them more relevant to participants who were not university students (see “Appendix A” in the “Supplemental materials” for the complete list of prompts). From this point onward in the experiment, participants’ apparent interactions with other participants were completely contrived.

### Essay writing and evaluation

To stage a transgression, we asked participants to write an essay on a topic that was personally important to them. Afterwards, participants received insulting feedback on their essay, ostensibly from one of the three other people in their four-person group^31,32^. For this procedure, participants first ranked the personal importance of several social issues. Then, we assigned all participants to write an essay for 5 min on the topic they ranked as most important. We used essays written by a separate sample of 41 Amazon Mechanical Turk workers to create the essays that the other three participants ostensibly wrote.

Following the writing task, participants read and evaluated the essays of the other three members of their four-person groups. We instructed participants to evaluate the three other participants’ essays on a number of criteria (e.g., logic, clarity) and to provide open-ended written feedback (see “Appendix A” of the “Supplemental materials”). During this part of the task, participants could not see which participants wrote which of the other three essays.

### Essay feedback and transgression

After participants evaluated the other three participants’ essays, we instructed them to review the feedback that the three other participants had provided on all four essays. Here, participants could see the identity of the participant who wrote each essay, a few lines of the essay (to remind them of context), the identity of each participant who evaluated the essay, and the complete feedback provided by each of the three evaluators (see “Supplemental materials” for examples). All of the feedback was politely delivered and neutral-to-positive, with one exception: Participants themselves received very strong negative feedback on their own essay from one of the other three participants: “I can’t believe an educated person would think like this. I hope this person learns a thing or two”.

The negative feedback and insult came either from the same participant with whom the participant interacted during the RCIT (Transgressor Value: High) or from one of the other two participants (Transgressor Value: Low).

### Apology manipulation

Following the essay writing, evaluation, and feedback tasks, we told participants that they would complete an upcoming task in pairs, and that we would randomly assign each participant to another partner. In reality, we always assigned participants to interact with the participant who had provided the negative feedback and insult (i.e., the transgressor). We then explained the rules of a standard Dictator Game^57^, which we simply called a Decision-Making Game. Here, we indicated that we would randomly assign one participant of the pair to be the Decision-Maker and the other participant to be the Recipient. The Decision-Maker would be able to send some, none, or all of his/her bonus earnings (up to $2.00 at this point in the experiment) to the Recipient. The Recipient’s role was simply to receive however much money the Decision-Maker sent. After completing a practice round of the Dictator Game from the perspective of both roles, we instructed participants that they had been randomly assigned to the Recipient role.

To manipulate apology, we told participants that they could exchange one message before beginning the Decision-Making Game. Decision-Makers (confederate participants in this case) always sent the first message, to which participants could respond. Participants in the apologetic message condition received a message that read “i'm really sorry i was mean about your essay. i want to send you some of my bonus to make it up to you” [sic]. Subsequently, the apologetic partner sent the participant $1.00. Participants in the neutral message condition simply received a message that read “this takes more concentration than i thought. at least it's more interesting than the last HIT i did” [sic]. (“HIT,” in the argot of Amazon’s Mechanical Turk, in an acronym for Human Intelligence Task).

### Partner preference task

The final task was a partner preference task, designed to measure whether participants preferred to continue working with the participant who had insulted their essay rather than with another participant. At this point, we told participants that they would participate in a subsequent task called a Trust Game. We did not give participants details regarding how to play the game, but we did tell them that they could choose to play the game either with the participant who had insulted their essay (and with whom they had just completed the Decision-Making Game) or with the participant who was neither their insulter nor RCIT partner. Due to a programming error, only the last 41% of participants (n = 399) completed the partner preference task.

### Measures

#### Perceived relationship value and exploitation risk

To confirm that the RCIT did indeed manipulate relationship value, we used the same four items as^26^ to measure participants’ feelings of closeness, similarity, liking, and desire to befriend each of the other three participants. As in^26^, we analyzed each item separately, while also comparing composite scores between ratings of RCIT partners (Cronbach’s α = 0.88) and non-RCIT partners (Cronbach’s α = 0.87).

We assessed participants’ perceptions of the insulter’s relationship value (e.g., “I feel that he/she could become an important person in my life”) and exploitation risk (e.g., “I would worry that he/she would take advantage of me”) with the 12-item Relationship Value and Exploitation Risk scale for Non-Close Others (RVEX-NCO; see “Appendix A”), based on^6^. Six items assess relationship value (RV); the other six assess participants’ perceptions of the transgressors’ exploitation risk (ER), defined as the likelihood that the transgressor will inflict costs on them in the future. Participants completed the RVEX-NCO at two time points: Once following the transgression manipulation, but before the apology manipulation (RV: McDonald’s *ω* = 0.945; ER: McDonald’s *ω* = 0.948), and once following the apology manipulation, but before the partner preference task (RV: McDonald’s *ω* = 0.950; ER: McDonald’s *ω* = 0.952). For brevity here, we report only results including the post-apology manipulation; analysis and interpretation of the model that includes post-transgression/pre-apology scores from the RVEX-NCO are available in the “Supplemental materials”. For the same reason—and because our main research hypothesis involved questions about the mediational role of perceived relationship value but not the mediational role of exploitation risk—we also omit results involving the Exploitation Risk subscale, which was significantly affected by apology (as in^3^), but not by manipulated relationship value or the interaction of apology and relationship value. A structural equation model that includes the Exploitation Risk measure also appears in the “Supplemental materials”.

Due to a programming error, participants responded to a random half of the questionnaire on one scale (5-point ordinal) and the other random half of the questionnaire on a different scale (9-point ordinal). Because there is no method for equating participants’ items on two sets of items with different response scales, we treated each participant’s responses on the 9-point ordinal scale as missing completely at random and handled the missing values using pairwise deletion with the WLSMV estimator (which was necessary for categorical outcomes). We combined the items for measuring relationship value and exploitation risk under the Item Response Theory framework, specifically using a graded response model for ordinal indicators^58^, which allows loadings and thresholds to vary across items. Syntax and output from the model selection procedures are available in “Supplemental materials”.

#### Forgiveness

Finally, to measure forgiveness, participants completed 17 questions from the Transgression-Related Interpersonal Motivations questionnaire for non-close others (TRIM-NCO^40,59^). Participants used a 5-point ordinal scale to indicate the extent to which they endorsed motivations toward the transgressor associated with benevolence (e.g., “I would act warmly towards him/her.”), avoidance (e.g., “I would avoid working with him/her”), and revenge (e.g., “I would want to get even with him/her.”; see “Appendix A” in the “Supplemental materials”). Following Forster et al. (in press), we modeled forgiveness using a bifactor (S-1) model in which forgiveness is modeled as a general factor (Hierarchical McDonald’s *ω* = 0.901) that suffuses all 17 items on the TRIM-NCO, along with two method factors, orthogonal to the general factor, that reflect item content related to the wording of the benevolence items and the revenge items^60–62^. We did not make any predictions regarding the two method factors, as they are forced in the bifactor (S-1) model to be orthogonal to the latent forgiveness factor^40^, and we did not report on any effects pertaining to the method factors (although they are reported in the “Supplemental materials”).

#### Suspicion probing and debriefing

After participants completed the TRIM-NCO, we used a funnel debriefing procedure to probe for suspicion^63^. The full procedure is available in the “Supplemental materials”.

## Supplementary Information

Supplementary Information.
