# Supplementary material for: Experimental evidence that apologies promote forgiveness by communicating relationship value
Source: Sci Rep. 2021 Jun 23;11:13107. doi: 10.1038/s41598-021-92373-y (PMC8222305; doi:10.1038/s41598-021-92373-y)
Supplement: Supplementary file 1 — Supplementary Information. [file 41598_2021_92373_MOESM1_ESM.pdf]

Experimental Evidence that Apologies Promote Forgiveness by Communicating Relationship Value

Supplemental Materials

Daniel E. Forster[1,2] Joseph Billingsley[3,2] Jeni L. Burnette[3]
Debra Lieberman[2] Yohsuke Ohtsubo[4,5] Michael E. McCullough[6,2,*]

[1] U.S. CCDC Army Research Laboratory
[2] University of Miami
[3] North Carolina State University
[4] Kobe University
[5] University of Tokyo
[6] University of California, San Diego

[*] Correspondence: Michael E. McCullough <memccullough@ucsd.edu>

Contents

Preregistration **3**
Study Information . . . 3
Sampling Plan . . . 3
Variables . . . 3
Design Plan . . . 4
Analysis Plan . . . 4

Suspicion Probing and Coding Procedures **6**
Questions . . . 6
Coding . . . 6

Descriptive Statistics **8**

Figures **10**
Post-Transgression RVEX . . . 10
Post-Apology RVEX, Forgiveness, and Partner Preference . . . 11

Manuscript Results **12**
Manipulation Check . . . 12
Path Analysis . . . 14

Supplemental Analyses **15**
Model Differences Between Suspicious and Non-Suspicious Participants . . . 15
Model Effects with Suspicious Participants Excluded . . . 16
Model Effects with All Participants . . . 18
Analysis of Essay Ratings . . . 21

Appendix A. Relationship Closeness Induction Task for Mturk Samples **22**

Appendix B. Items for Providing Feedback on Essays **23**

Appendix C. Relationship Value and Exploitation Risk scale for Non-Close Others (RVEX-NCO) **24**

Appendix D. Transgression Related Interpsonal Motivations Inventory for Non-Close Others (TRIM-NCO) **25**

Preregistration

This experiment was preregistered on the Open Science Framework (OSF). To preserve anonymity during the review process, we repeat the content from the preregistration forms here without author information.

Study Information

Title: The causal influence of relationship value on forgiveness

Research Questions: Does relationship closeness increase forgiveness following a transgression?

Hypothesis: We hypothesize that people who highly value a relationship partner will be more forgiving after a transgression than people who do not highly value a relationship partner.

Sampling Plan

Existing Data: Registration prior to creation of data.

Explanation of existing data: There has been no data collection for this study.

Data collection procedures: Data will be collected using Amazon's Mechanical Turk (Mturk) via SoPHIE (Software Platform for Human Interaction Experiments). Participants will be promised a baseline payment of $1.00 and the opportunity to earn bonus money. Since the study uses deception, all subjects who complete the study will be paid the same amount (between $7.50, for an estimated 45-minute completion time, and $10.00, for a 1-hour estimated completion time). Participants will be restricted to subjects from the U.S. who have at least a 95% approval rate from Mturk. Additionally, subjects who have participated in our previous experiments on Mturk, which have used some of the same critical elements of the design, will also be excluded from participating. Once data collection commences, completion should take approximately 1-2 weeks.

Sample size: The sample size for this study is 100 subjects per condition in a 2 (reconciliation: apology v. neutral) x 2 (relationship: strong v. weak) between-subjects design. In total, we will need 400 "clean" subjects (i.e., subjects who completed all parts of the experiment and did not indicate any suspicion).

Sample size rationale: In our previous work on the effects of apologies on forgiveness, we collected 100 subjects per condition. Our results from that experiment were very robust, so we decided to collect 100 subjects per condition for this experiment, as well.

Stopping rule: We will stop when we can confidently determine that we have enough "clean" subjects. Responses to suspicion probes will be the only data analyzed during data collection.

Variables

Manipulated variables: We will manipulate whether subjects are insulted by a person with whom they interacted during the Relationship Closeness Induction Task (RCIT; see attached). The timing for each section of the RCIT will depend on how people perform on the task in pilot data sessions.

We will also manipulate whether subjects receive an apologetic message (with an offer of compensation) or a neutral message from the transgressor. The apology will read as, "i'm really sorry i was mean about your essay. i want to send you some of my bonus to make it up to you," whereas the neutral message will read, "this takes more concentration than i thought. at least it's more interesting than the last HIT i did" [sic].

Measured variables:

- Demographics (Age, Sex, Ethnicity, Race)
- RCIT Manipulation Check (3 targets; see attached)

- Relationship Value and Exploitation Risk (3 time points; see attached)
- Transgression-Related Interpersonal Motivations (see attached)
- With whom the subject decides to interact in a subsequent task

[**Note: We preregistered three time points, but we only collected two. The first measure of RVEX was inadvertently dropped during the programming of the experiment.**]

Indices: Relationship value and Exploitation Risk is a two-factor model, each with six indicators. These will be measured as latent variables.

Transgression-Related Interpersonal Motivations (TRIM) will be measured using Rasch modeling procedures, which has the property of creating a continuous ratio-scale metric from Likert-type items (using Item Response Theory). This procedure has been used to model TRIM data in the past.

Amount sent in the Trust Game will be treated either as a count variable (poisson, negative binomial, or their zero-inflated variations) or a continuous variable, depending on which model results in the smallest loss of information (according to BIC).

[**NOTE: The mention of the Trust Game was an uncorrected artifact of copying our other preregistrations on this topic. The Trust Game was not part of this experiment, which is why it was not mentioned in the 'Measured variables' section above.**]

Design Plan

Study type: Experiment - A researcher randomly assigns treatments to study subjects, this includes field or lab experiments. This is also known as an intervention experiment and includes randomized controlled trials.

Blinding: For studies that involve human subjects, they will not know the treatment group to which they have been assigned.,Research personnel who interact directly with the study subjects (either human or non-human subjects) will not be aware of the assigned treatments.

Study design: This is a 2 (closeness: RCIT v. No RCIT) x 2 (message: apologetic v. neutral) between-subjects factorial design.

Randomization: We will not be randomly sampling, but we will use random assignment across our conditions. Participants are assigned to their conditions based on their subject numbers (e.g., Participant 1 gets Condition 1, S2 gets C2, S3 gets C3, S4 gets C4, S5 gets C1, S6 gets C2, etc.). Data collection on Mturk is rapid enough that subjects assigned to condition 1 will not be any less likely to have been assigned to any other condition. Further, SoPHIE's system for assigning subject numbers has a seemingly random pattern, which means that the first person to enter the experiment is not necessarily going to be assigned the first label.

Analysis Plan

Statistical Models: We will specify structural equation models using the lavaan package in R to conduct all of the central analyses.

MANIPULATION CHECK: Does RCIT increase closeness?

We will create a single factor from the four manipulation check items to form a "relationship closeness" latent variable. We will assess the latent mean difference between respondents' relationship closeness toward those with whom they interacted in the RCIT and those with whom they did not interact in the RCIT.

MODEL 1: Does RCIT, apologies, or their interaction influence relationship value and exploitation risk following a transgression?

Latent variables for Relationship Value and Exploitation Risk (at the time following the insult) will be predicted by the effect-coded RCIT variable (RCIT = 1; No RCIT = -1), effect-coded apology (Apology = 1;

No Apology = -1), and their interaction. If the interaction does not predict relationship value or exploitation risk, then the interaction term will be dropped from the model and only the main effects will be interpreted.

MODEL 2a: Does relationship closeness interact with apologies to predict forgiveness, as measured by TRIM?

TRIM will be predicted by effect-coded RCIT (RCIT = 1; No RCIT = -1), apology (apology = 1; neutral = -1), and their interaction. From this model, we will either retain a main effects only model or an interaction model.

MODEL 2b: Does relationship closeness interact with apologies to predict forgiveness, as measured by Partner Choice (PC)?

This model is identical to Model 3a, except TRIM is replaced by the categorical outcome of whether the subject decided to retain (PC = 1) or reject (PC = 0) their current partner.

MODEL 3a: Do Relationship Value and Exploitation Risk mediate the relationships between RCIT/apology (and their interaction, if applicable) and forgiveness, as measured by TRIM?

This model will be identical to the final Model 2a, except that Relationship Value and Exploitation Risk latent variables will be included as mediators.

MODEL 3b: Do Relationship Value and Exploitation Risk mediate the relationships between RCIT/apology (and their interaction, if applicable) and forgiveness, as measured by PC?

This model is identical to Model 2b, except TRIM is replaced by the categorical outcome of whether the subject decided to retain (PC = 1) or reject (PC = 0) their current partner.

Transformations: We will use effect coding for our independent variables when analyzing main effects/interactions.

Model fit for Rasch modeling will be assessed using the mixRasch package in R. We will extract factor scores from our Rasch model and include their values as observations in structural equation models specified using the lavaan package in R.

Follow-up analyses: If the interaction between apology and RCIT is significant, we will test whether apologies only influence forgiveness (as measured by TRIM and Partner Choice, separately) when coming from someone with whom the subject has previously interacted. We have no directional hypotheses regarding the simple effects of this interaction.

Inference Criteria: We will largely ignore Chi-Square model fit, which is known to be very sensitive to large sample sizes (we will still assess residuals to ensure that rejection of the exact-fit hypothesis is due to its sensitivity to sample size, rather than a true discrepancy between our model and our data). Instead, we will focus our attention on meeting acceptable criteria for CFI, RMSEA, and SRMR, which are less sensitive to detecting minor discrepancies between models/data with larger sample sizes.

We will assess the evidence of relationships among variables using p-values derived from Maximum Likelihood estimation. For mediation analyses, standard errors will be estimated using bootstrapping.

If we encounter alternative models that cannot be directly assessed with exact fit indices, we will use BIC to select the model with the smallest loss of information.

Data exclusion: Data will only be excluded if (1) subjects indicated that they were suspicious of the deception in our experimental design (e.g., they did not believe that they were interacting with a real person) or (2) subjects somehow by-passed our exclusion criteria, namely if they had participated in a previous experiment of ours or if they had participated in this study twice (for the latter case, we will only exclude their second case, as long as they did not indicate any suspicion in the first case).

Missing data: Missing data will be handled with Full Information Maximum Likelihood estimation (FIML). If FIML is unavailable for any analysis, we will use list-wise deletion; however, as far as we know, FIML is available for all model estimation types that we are interested in.

Exploratory analysis: N/A

Suspicion Probing and Coding Procedures

These questions were asked after subjects completed all of the procedures in the experiment.

Questions

1. Do you have any questions?
   a. If yes, please enter any questions you might have.
2. Was the experiment clear? Did all aspects of the procedure make sense?
   a. If yes, please explain what did not make sense during the experiment.
3. Do you have any personal feelings or reactions to the experiment?
   a. If yes, please describe your personal feelings and reactions to the experiment.
4. Today's experiment was designed to help us test some very specific hypotheses about human behavior. Do you have any idea what those hypotheses were?
   a. If yes, please describe what you think our hypotheses are.
5. Did you find any aspect of the procedure odd, upsetting, or disturbing?
   a. If yes, please explain which aspects of the procedure you found odd, upsetting, or disturbing?
6. Did you wonder at any point whether there was more than meets the eye to any of the procedures that we had you complete today? That is, do you think that there may have been any information that we held back from explaining to you?
   a. If yes, please explain what information, if any, you believe we may have held back from you.
   b. If you mentioned anything in response to the previous question, at what point during the study did you begin to think about what you mentioned? Also, how (if at all) do you think those aspects of the study affected your behavior?

Coding

These instructions were provided to research assistants who coded for suspicion. For analysis, "hard" and "soft" suspicion were grouped together as "suspicious."

Hard Suspicion:

1. The person indicates that they believe the other person/people was/were not real.
2. The person indicates that they believe the other person/people received different sets of instructions (or was told what to say/do).
3. The person indicates that the study was designed to determine whether they are forgiving.
4. The person indicates that the study was designed to determine how they would respond to unfairness/unkindness.

~ These people use language like, "I believe that...," "The other person was (not)..."

Soft Suspicion:

1. The person was not sure whether the other person/people was/were real.
2. The person was not sure whether the other person/people received different instructions.

3. After indicating they don't believe the other person/people were real, they indicate (under Probe 7) that they only thought of it after they were asked.

~ These people use language like, "I'm not sure if. . . ," "I wonder(ed) if. . . ," "I thought that maybe. . . "

No Suspicion:

1. The person writes nothing in the free response section.
2. The person is "suspicious" about something other than the authenticity of the other people. e.g., "I wonder why the other person was so mean," "Why would you ask us those types of questions/give us those types of tasks?"
3. The person is vague about suspicions. e.g., "These studies usually hold something back," "Researchers don't always want us to know the research questions."

Descriptive Statistics

Table 1: Descriptives Post-Transgression Manipulation

		value	N	Missing	Mean	SD	Min	Q1	Median	Q3	Max
1	rv1	High-Value	496	1	0.04	0.96	-2.35	-0.60	0.05	0.74	2.36
1.1		Low-Value	474	0	-0.21	0.82	-2.19	-0.76	-0.13	0.27	2.24
2	er1	High-Value	496	1	0.02	0.86	-1.71	-0.59	0.06	0.57	2.41
2.1		Low-Value	474	0	0.21	0.82	-2.49	-0.28	0.25	0.68	2.75

Table 2: Descriptives Post-Apology Manipulation

		value_apology	N	Missing	Mean	SD	Min	Q1	Median	Q3	Max
1	rv2	High-Value/Apology	238	0	0.39	0.91	-2.17	-0.09	0.46	1.02	2.34
1.1		High-Value/No-Apology	258	1	0.01	1.04	-2.42	-0.62	0.06	0.68	2.34
1.2		Low-Value/Apology	234	0	0.36	0.83	-2.48	-0.09	0.23	0.89	2.29
1.3		Low-Value/Non-Apology	240	0	-0.33	0.86	-2.41	-0.90	-0.21	0.26	2.11
2	er2	High-Value/Apology	238	0	-0.31	0.84	-1.88	-0.94	-0.27	0.22	2.36
2.1		High-Value/No-Apology	258	1	0.08	1.01	-1.96	-0.69	0.09	0.75	2.23
2.2		Low-Value/Apology	234	0	-0.21	0.80	-2.11	-0.76	-0.18	0.28	2.44
2.3		Low-Value/Non-Apology	240	0	0.25	0.96	-2.63	-0.43	0.28	0.91	2.66
3	gen	High-Value/Apology	238	0	0.18	0.83	-2.25	-0.38	0.19	0.76	1.40
3.1		High-Value/No-Apology	257	2	-0.05	0.93	-2.36	-0.60	-0.02	0.64	1.40
3.2		Low-Value/Apology	232	2	0.11	0.77	-2.34	-0.40	0.10	0.62	1.40
3.3		Low-Value/Non-Apology	238	2	-0.38	0.83	-2.36	-0.94	-0.42	0.22	1.40
4	PSwitch	High-Value/Apology	104	134	0.31	0.46	0.00	0.00	0.00	1.00	1.00
4.1		High-Value/No-Apology	105	154	0.37	0.49	0.00	0.00	0.00	1.00	1.00
4.2		Low-Value/Apology	96	138	0.33	0.47	0.00	0.00	0.00	1.00	1.00
4.3		Low-Value/Non-Apology	94	146	0.48	0.50	0.00	0.00	0.00	1.00	1.00

Table 3: Correlations Among Outcomes

	rv1	er1	rv2	er2	gen	PSwitch
rv1	1					
er1	-0.451	1				
rv2	0.761	-0.318	1			
er2	-0.376	0.736	-0.576	1		
gen	0.597	-0.506	0.751	-0.68	1	
PSwitch	-0.349	0.183	-0.478	0.348	-0.507	1

Table 4: McDonald's Omega for Self-Report Scales

Factor	Omega
rv2_omega	0.9452587
er2_omega	0.9478250
rv3_omega	0.9497210
er3_omega	0.9521374
trim_omega_h	0.9005291
trim_omega_hb	0.2848261
trim_omega_hr	0.5716998

Figures

Post-Transgression RVEX

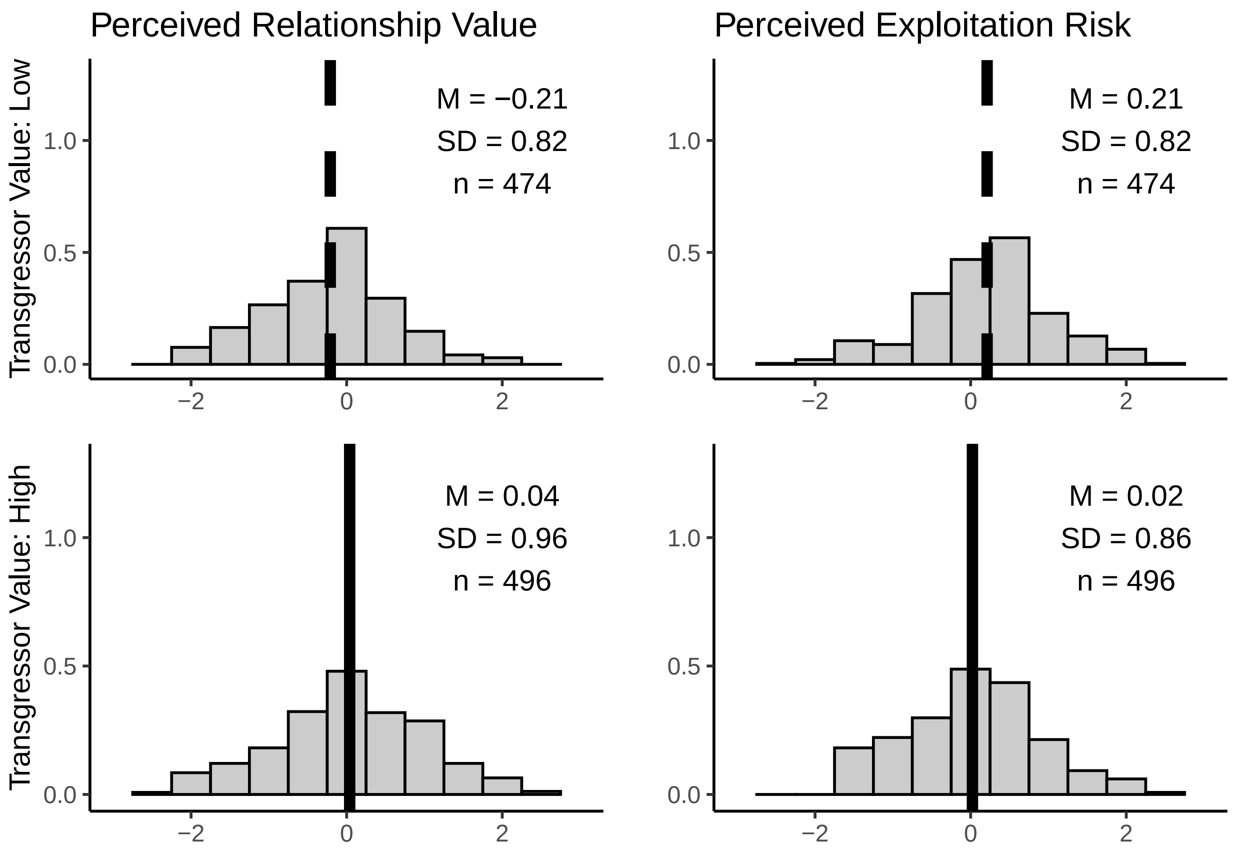

Post-Apology RVEX, Forgiveness, and Partner Preference

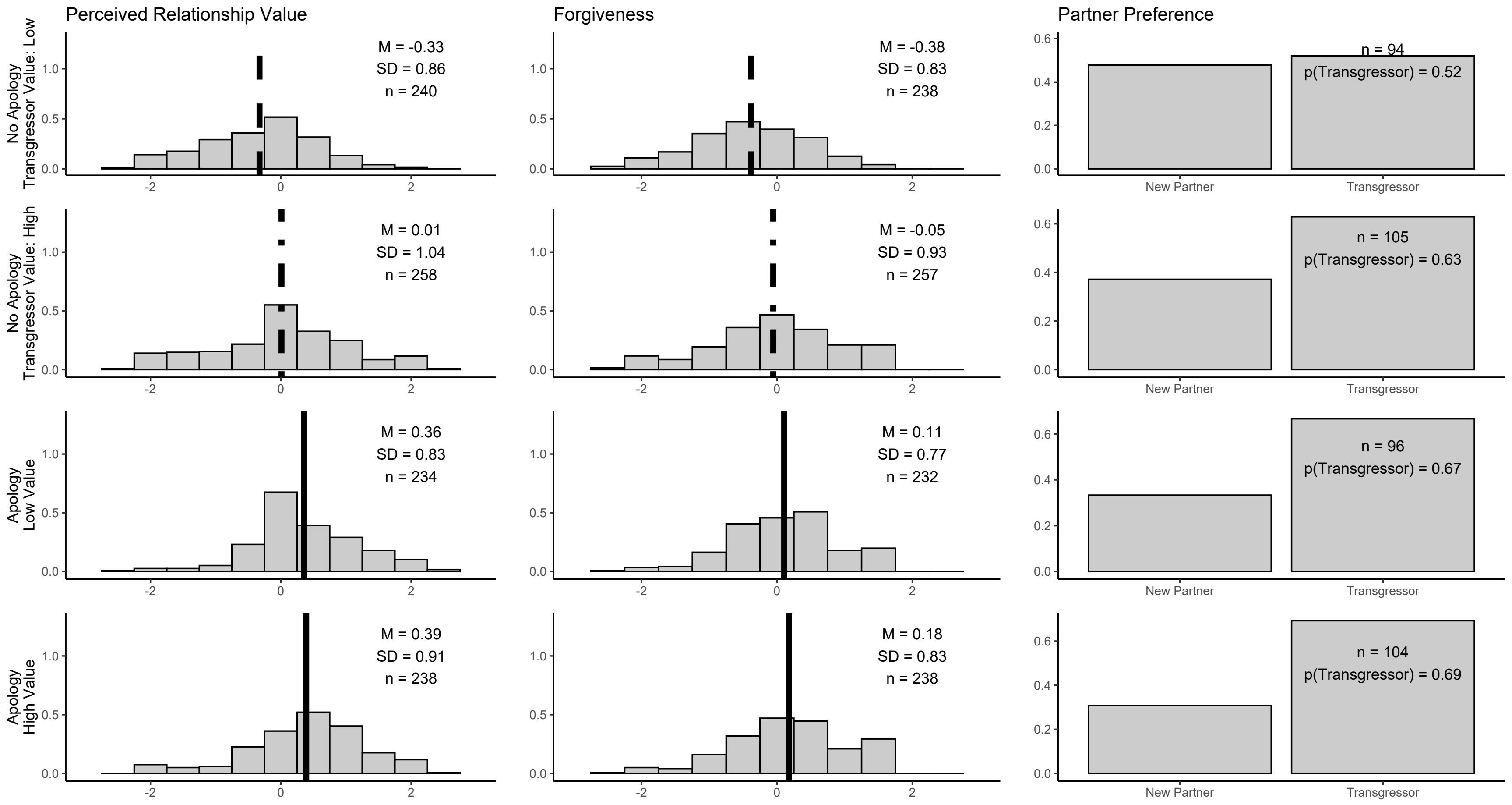

Manuscript Results

Manipulation Check

Reliability Analysis for Closeness Ratings of RCIT Partner

```
##
## Reliability analysis
## Call: psych::alpha(x = rcit_data[, c("rcit_close", "rcit_friends",
##     "rcit_similar", "rcit_like")])
##
##   raw_alpha std.alpha G6(smc) average_r S/N    ase mean  sd median_r
##       0.88      0.88    0.86      0.65 7.4 0.0064  6.1 1.6     0.63
##
##  lower alpha upper     95% confidence boundaries
## 0.87 0.88 0.89
##
##  Reliability if an item is dropped:
##              raw_alpha std.alpha G6(smc) average_r S/N alpha se   var.r med.r
## rcit_close        0.86      0.86    0.82      0.68 6.2   0.0080 0.00635  0.67
## rcit_friends      0.82      0.83    0.76      0.61 4.8   0.0096 0.00038  0.61
## rcit_similar      0.85      0.86    0.81      0.67 6.0   0.0084 0.00672  0.63
## rcit_like         0.84      0.85    0.78      0.65 5.5   0.0086 0.00038  0.64
##
##  Item statistics
##                n raw.r std.r r.cor r.drop mean  sd
## rcit_close   971  0.84  0.84  0.75   0.71  5.4 2.0
## rcit_friends 971  0.89  0.89  0.86   0.79  6.6 1.9
## rcit_similar 971  0.85  0.85  0.77   0.72  5.7 2.0
## rcit_like    971  0.85  0.86  0.81   0.75  6.9 1.6
##
## Non missing response frequency for each item
##                 1    2    3    4    5    6    7    8    9 miss
## rcit_close   0.06 0.04 0.09 0.09 0.18 0.22 0.18 0.08 0.05    0
## rcit_friends 0.02 0.02 0.03 0.04 0.14 0.17 0.25 0.15 0.17    0
## rcit_similar 0.04 0.04 0.07 0.10 0.16 0.18 0.23 0.10 0.07    0
## rcit_like    0.01 0.00 0.02 0.03 0.10 0.18 0.26 0.18 0.20    0
```

Reliability Analysis for Closeness Ratings of non-RCIT Group Members

```
##
## Reliability analysis
## Call: psych::alpha(x = rcit_data[, c("other_close", "other_friends",
##     "other_similar", "other_like")])
##
##   raw_alpha std.alpha G6(smc) average_r S/N    ase mean  sd median_r
##       0.87      0.87    0.86      0.63 6.8 0.0069  3.5 1.5     0.64
##
##  lower alpha upper     95% confidence boundaries
## 0.86 0.87 0.88
##
##  Reliability if an item is dropped:
##               raw_alpha std.alpha G6(smc) average_r S/N alpha se  var.r med.r
## other_close        0.86      0.86    0.82      0.68 6.4   0.0078 0.0060  0.64
## other_friends      0.83      0.83    0.78      0.63 5.0   0.0091 0.0094  0.64
## other_similar      0.81      0.82    0.78      0.60 4.5   0.0107 0.0222  0.52
## other_like         0.83      0.83    0.78      0.62 4.8   0.0095 0.0119  0.63
##
##  Item statistics
##                 n raw.r std.r r.cor r.drop mean  sd
## other_close   971  0.81  0.81  0.72   0.66  2.6 1.8
## other_friends 971  0.85  0.85  0.80   0.73  4.1 1.8
## other_similar 971  0.89  0.88  0.83   0.78  3.2 1.9
## other_like    971  0.85  0.86  0.81   0.74  4.2 1.7
```

Table 5: Effect of RCIT on Closeness Measures

outcome	Mean Difference	se	t(df)	p.value	Hedge's g	g_LB	g_UB
Close	2.847065	0.0703697	40.459 (970)	$< .001$	1.482022	1.377961	1.586082
Friends	2.455201	0.0656857	37.378 (970)	$< .001$	1.348236	1.250500	1.445973
Like	2.749228	0.0649411	42.334 (970)	$< .001$	1.657855	1.539513	1.776196
Similar	2.419156	0.0767622	31.515 (970)	$< .001$	1.237083	1.134802	1.339365
Composite Score	2.617662	0.0574267	45.583 (970)	$< .001$	1.666045	1.555278	1.776812

Path Analysis

Table 6: Main Model Analyses Reported in Manuscript

outcome	path	pred	est	se	est_se	pval	low2.5	up2.5
RV3	Direct	INS_EFF	0.118	0.036	3.332	0.001	0.049	0.188
RV3	Direct	AP_EFF	0.366	0.038	9.749	0.000	0.293	0.440
RV3	Direct	AP_INT	-0.092	0.036	-2.538	0.011	-0.164	-0.021
PSWITCH	Direct	INS_EFF	-0.014	0.064	-0.214	0.831	-0.139	0.112
PSWITCH	Total	INS_EFF	-0.087	0.066	-1.310	0.190	-0.216	0.043
PSWITCH	Total indirect	INS_EFF	-0.073	0.022	-3.290	0.001	-0.116	-0.029
PSWITCH	Direct	AP_EFF	0.088	0.066	1.328	0.184	-0.042	0.218
PSWITCH	Total	AP_EFF	-0.138	0.066	-2.079	0.038	-0.268	-0.008
PSWITCH	Total indirect	AP_EFF	-0.226	0.028	-7.936	0.000	-0.282	-0.170
PSWITCH	Direct	AP_INT	-0.006	0.063	-0.098	0.922	-0.129	0.117
PSWITCH	Total	AP_INT	0.051	0.066	0.774	0.439	-0.078	0.179
PSWITCH	Total indirect	AP_INT	0.057	0.023	2.484	0.013	0.012	0.102
PSWITCH	Direct	RV3	-0.616	0.049	-12.489	0.000	-0.713	-0.520
GEN	Direct	INS_EFF	0.017	0.022	0.747	0.455	-0.027	0.061
GEN	Total	INS_EFF	0.109	0.032	3.405	0.001	0.046	0.171
GEN	Total indirect	INS_EFF	0.092	0.028	3.336	0.001	0.038	0.146
GEN	Direct	AP_EFF	-0.087	0.023	-3.703	0.000	-0.133	-0.041
GEN	Total	AP_EFF	0.198	0.032	6.199	0.000	0.135	0.260
GEN	Total indirect	AP_EFF	0.285	0.029	9.790	0.000	0.228	0.342
GEN	Direct	AP_INT	0.002	0.023	0.095	0.924	-0.042	0.046
GEN	Total	AP_INT	-0.070	0.031	-2.242	0.025	-0.130	-0.009
GEN	Total indirect	AP_INT	-0.072	0.028	-2.545	0.011	-0.127	-0.016
GEN	Direct	RV3	0.776	0.018	42.596	0.000	0.741	0.812
BENEV	Direct	INS_EFF	-0.026	0.016	-1.608	0.108	-0.058	0.006
BENEV	Direct	AP_EFF	0.044	0.017	2.531	0.011	0.010	0.078
BENEV	Direct	AP_INT	-0.001	0.016	-0.078	0.937	-0.033	0.030
BENEV	Direct	RV3	0.111	0.047	2.368	0.018	0.019	0.202
REV	Direct	INS_EFF	0.021	0.033	0.627	0.531	-0.044	0.086
REV	Direct	AP_EFF	0.149	0.038	3.974	0.000	0.076	0.223
REV	Direct	AP_INT	0.041	0.034	1.223	0.221	-0.025	0.107
REV	Direct	RV3	-0.374	0.094	-3.981	0.000	-0.558	-0.190
AP_EFF	RV3	GEN	0.285	0.029	9.790	0.000	0.228	0.342
AP_EFF	RV3	PSWITCH	-0.226	0.028	-7.936	0.000	-0.282	-0.170
AP_INT	RV3	GEN	-0.072	0.028	-2.545	0.011	-0.127	-0.016
AP_INT	RV3	PSWITCH	0.057	0.023	2.484	0.013	0.012	0.102
INS_EFF	RV3	GEN	0.092	0.028	3.336	0.001	0.038	0.146
INS_EFF	RV3	PSWITCH	-0.073	0.022	-3.290	0.001	-0.116	-0.029

Supplemental Analyses

Model Differences Between Suspicious and Non-Suspicious Participants

Table 7: Comparison of Effects for Suspicious and Non-Suspicious Participants

regression	s_est_se	n_est_se	d_est_se	d_p
RV3.ON.RV2	0.784 (0.096)	0.752 (0.081)	0.032 (0.126)	0.800
RV3.ON.ER2	-0.007 (0.123)	-0.018 (0.075)	0.011 (0.142)	0.940
ER3.ON.ER2	0.8 (0.082)	0.813 (0.129)	-0.013 (0.154)	0.933
ER3.ON.RV2	-0.05 (0.082)	-0.082 (0.096)	0.031 (0.124)	0.801
GEN.ON.RV3	0.559 (0.08)	0.53 (0.069)	0.029 (0.086)	0.731
GEN.ON.ER3	-0.389 (0.081)	-0.365 (0.073)	-0.024 (0.08)	0.763
BENEV.ON.RV3	0.115 (0.063)	0.052 (0.055)	0.064 (0.066)	0.335
BENEV.ON.ER3	0.092 (0.054)	0.068 (0.043)	0.024 (0.058)	0.673
REV.ON.RV3	-0.379 (0.145)	-0.502 (0.115)	0.123 (0.149)	0.412
REV.ON.ER3	-0.004 (0.109)	-0.117 (0.096)	0.113 (0.134)	0.400
RV2.ON.INS_EFF	0.121 (0.056)	0.148 (0.053)	-0.027 (0.075)	0.721
ER2.ON.INS_EFF	-0.122 (0.058)	-0.118 (0.057)	-0.005 (0.079)	0.950
RV3.ON.INS_EFF	-0.007 (0.047)	0.013 (0.047)	-0.02 (0.066)	0.762
RV3.ON.AP_EFF	0.334 (0.055)	0.356 (0.057)	-0.022 (0.078)	0.776
RV3.ON.AP_INT	-0.043 (0.055)	-0.169 (0.056)	0.126 (0.076)	0.097
ER3.ON.INS_EFF	0.046 (0.055)	0.009 (0.07)	0.037 (0.093)	0.688
ER3.ON.AP_EFF	-0.249 (0.066)	-0.295 (0.077)	0.046 (0.093)	0.620
ER3.ON.AP_INT	0.045 (0.062)	-0.036 (0.07)	0.081 (0.093)	0.384
GEN.ON.INS_EFF	0.023 (0.029)	0.037 (0.029)	-0.014 (0.041)	0.737
GEN.ON.AP_EFF	-0.083 (0.03)	-0.09 (0.03)	0.007 (0.042)	0.866
GEN.ON.AP_INT	-0.038 (0.03)	0.014 (0.031)	-0.051 (0.043)	0.235
BENEV.ON.INS_EFF	-0.045 (0.024)	-0.011 (0.027)	-0.035 (0.035)	0.322
BENEV.ON.AP_EFF	0.031 (0.027)	0.087 (0.031)	-0.057 (0.039)	0.143
BENEV.ON.AP_INT	-0.006 (0.024)	0.013 (0.027)	-0.019 (0.036)	0.591
REV.ON.INS_EFF	-0.013 (0.057)	0.039 (0.05)	-0.052 (0.077)	0.504
REV.ON.AP_EFF	0.178 (0.062)	0.104 (0.056)	0.073 (0.079)	0.352
REV.ON.AP_INT	0.066 (0.054)	-0.006 (0.052)	0.072 (0.076)	0.344
PSWITCH.ON.RV3	-0.631 (0.123)	-0.425 (0.096)	-0.206 (0.152)	0.175
PSWITCH.ON.ER3	0.116 (0.127)	0.131 (0.1)	-0.016 (0.156)	0.920
PSWITCH.ON.INS_EFF	-0.164 (0.093)	0.093 (0.089)	-0.257 (0.129)	0.047
PSWITCH.ON.AP_EFF	-0.01 (0.102)	0.148 (0.091)	-0.158 (0.136)	0.246
PSWITCH.ON.AP_INT	0.042 (0.096)	-0.023 (0.085)	0.065 (0.127)	0.610

In the previous table, we tested whether paths differed between suspicious and non-suspicious participants. Of the thirty-two paths tested in the model, only one path differed significantly between suspicious and non-suspicious participants. The path in question was the direct path from the effect of whether the insulter was the RCIT partner on participants' desire to change interaction partners. Although neither suspicious (b = -0.164, p = .082) nor non-suspicious participants (b = 0.089, p = .307) yielded a significant effect, the two groups differed from each other, z = -1.968, p = .049. Because this effect was inconsequential, and because we would expect to find one significant difference among twenty truly non-significant differences, we opted to include all participants in the analyses reported in the manuscript. Here, in this supplemental document, we also present analyses with suspicious participants excluded.

Note that the model under which we compared suspicious and non-suspicious paths has additional paths compared to the model reported in the manuscript. First, the comparison analysis include perceptions of exploitation risk as a mediator. Second, the comparison analysis includes perceptions of relationship value and exploitation risk immediately following the transgression, but before the apology. The model we reported in the manuscript helped us limit the scope to address specific research questions, whereas the model reported here gives a more thorough analysis for interested readers.

Model Effects with Suspicious Participants Excluded

Per our preregistration, we also conducted our analyses after excluding all participants who exhibited suspicion.

Table 8: Main Model Analyses w/ Suspicious Participants Excluded

outcome	path	pred	est	se	est_se	pval	low2.5	up2.5
RV2	Direct	INS_EFF	0.139	0.054	2.591	0.010	0.034	0.245
ER2	Direct	INS_EFF	-0.121	0.055	-2.190	0.028	-0.230	-0.013
RV3	Direct	RV2	0.733	0.033	22.373	0.000	0.668	0.797
RV3	Direct	INS_EFF	0.012	0.047	0.261	0.794	-0.080	0.105
RV3	Direct	AP_EFF	0.324	0.052	6.195	0.000	0.222	0.427
RV3	Direct	AP_INT	-0.162	0.052	-3.109	0.002	-0.263	-0.060
ER3	Direct	ER2	0.713	0.042	16.841	0.000	0.630	0.796
ER3	Direct	INS_EFF	0.006	0.057	0.107	0.915	-0.105	0.117
ER3	Direct	AP_EFF	-0.233	0.060	-3.906	0.000	-0.350	-0.116
ER3	Direct	AP_INT	-0.035	0.058	-0.594	0.552	-0.149	0.080
PSWITCH	Direct	INS_EFF	0.093	0.085	1.095	0.273	-0.073	0.258
PSWITCH	Total	INS_EFF	0.026	0.089	0.296	0.767	-0.147	0.200
PSWITCH	Total indirect	INS_EFF	-0.066	0.027	-2.477	0.013	-0.119	-0.014
PSWITCH	Direct	AP_EFF	0.147	0.086	1.707	0.088	-0.022	0.316
PSWITCH	Total	AP_EFF	-0.042	0.089	-0.475	0.635	-0.216	0.132
PSWITCH	Total indirect	AP_EFF	-0.189	0.033	-5.655	0.000	-0.255	-0.124
PSWITCH	Direct	AP_INT	-0.021	0.085	-0.251	0.802	-0.188	0.145
PSWITCH	Total	AP_INT	0.044	0.089	0.501	0.617	-0.129	0.218
PSWITCH	Total indirect	AP_INT	0.066	0.029	2.285	0.022	0.009	0.122
PSWITCH	Direct	RV3	-0.447	0.078	-5.763	0.000	-0.600	-0.295
PSWITCH	Direct	ER3	0.189	0.090	2.115	0.034	0.014	0.365
GEN	Direct	INS_EFF	0.035	0.028	1.246	0.213	-0.020	0.089
GEN	Total	INS_EFF	0.134	0.044	3.086	0.002	0.049	0.220
GEN	Total indirect	INS_EFF	0.100	0.039	2.576	0.010	0.024	0.176
GEN	Direct	AP_EFF	-0.087	0.028	-3.109	0.002	-0.142	-0.032
GEN	Total	AP_EFF	0.198	0.044	4.507	0.000	0.112	0.284

Table 8: Main Model Analyses w/ Suspicious Participants Excluded *(continued)*

outcome	path	pred	est	se	est_se	pval	low2.5	up2.5
GEN	Total indirect	AP_EFF	0.285	0.039	7.350	0.000	0.209	0.361
GEN	Direct	AP_INT	0.014	0.027	0.524	0.601	-0.039	0.067
GEN	Total	AP_INT	-0.060	0.044	-1.377	0.169	-0.145	0.025
GEN	Total indirect	AP_INT	-0.074	0.038	-1.932	0.053	-0.149	0.001
GEN	Direct	RV3	0.556	0.036	15.639	0.000	0.486	0.625
GEN	Direct	ER3	-0.450	0.035	-12.710	0.000	-0.519	-0.380
BENEV	Direct	INS_EFF	-0.011	0.027	-0.394	0.693	-0.063	0.042
BENEV	Direct	AP_EFF	0.086	0.028	3.032	0.002	0.030	0.141
BENEV	Direct	AP_INT	0.012	0.027	0.438	0.661	-0.041	0.064
BENEV	Direct	RV3	0.062	0.045	1.390	0.164	-0.026	0.150
BENEV	Direct	ER3	0.063	0.042	1.494	0.135	-0.020	0.145
REV	Direct	INS_EFF	0.037	0.048	0.767	0.443	-0.058	0.132
REV	Direct	AP_EFF	0.103	0.053	1.933	0.053	-0.001	0.208
REV	Direct	AP_INT	-0.003	0.048	-0.057	0.954	-0.097	0.092
REV	Direct	RV3	-0.502	0.086	-5.810	0.000	-0.671	-0.333
REV	Direct	ER3	-0.075	0.073	-1.019	0.308	-0.219	0.069
AP_EFF	ER3	GEN	0.105	0.028	3.711	0.000	0.049	0.160
AP_EFF	RV3	GEN	0.180	0.031	5.877	0.000	0.120	0.240
AP_EFF	ER3	PSWITCH	-0.044	0.024	-1.818	0.069	-0.092	0.003
AP_EFF	RV3	PSWITCH	-0.145	0.034	-4.255	0.000	-0.212	-0.078
AP_INT	ER3	GEN	0.016	0.026	0.596	0.551	-0.036	0.067
AP_INT	RV3	GEN	-0.090	0.029	-3.116	0.002	-0.146	-0.033
AP_INT	ER3	PSWITCH	-0.007	0.011	-0.571	0.568	-0.029	0.016
AP_INT	RV3	PSWITCH	0.072	0.026	2.781	0.005	0.021	0.123
INS_EFF	ER3	GEN	-0.003	0.026	-0.107	0.915	-0.053	0.047
INS_EFF	ER3.ER2	GEN	0.039	0.018	2.146	0.032	0.003	0.075
INS_EFF	RV3	GEN	0.007	0.026	0.261	0.794	-0.045	0.058
INS_EFF	RV3.RV2	GEN	0.057	0.022	2.567	0.010	0.013	0.100
INS_EFF	ER3	PSWITCH	0.001	0.011	0.107	0.915	-0.020	0.022
INS_EFF	ER3.ER2	PSWITCH	-0.016	0.011	-1.539	0.124	-0.037	0.004
INS_EFF	RV3	PSWITCH	-0.006	0.021	-0.262	0.794	-0.047	0.036
INS_EFF	RV3.RV2	PSWITCH	-0.046	0.019	-2.418	0.016	-0.083	-0.009

Model Effects with All Participants

Table 9: Main Model Analyses w/ All Participants

outcome	path	pred	est	se	est_se	pval	low2.5	up2.5
RV2	Direct	INS_EFF	0.236	0.053	4.449	0.000	0.132	0.339
RV3	Direct	RV2	0.758	0.030	25.147	0.000	0.699	0.817
RV3	Direct	INS_EFF	0.010	0.047	0.215	0.829	-0.082	0.103
RV3	Total	INS_EFF	0.189	0.056	3.365	0.001	0.079	0.299
RV3	Total indirect	INS_EFF	0.179	0.041	4.332	0.000	0.098	0.259
RV3	Direct	AP_EFF	0.553	0.059	9.440	0.000	0.438	0.668
RV3	Direct	AP_INT	-0.142	0.056	-2.532	0.011	-0.251	-0.032
PSWITCH	Direct	INS_EFF	-0.014	0.060	-0.229	0.819	-0.132	0.104
PSWITCH	Total	INS_EFF	-0.089	0.064	-1.398	0.162	-0.214	0.036
PSWITCH	Total indirect	INS_EFF	-0.075	0.022	-3.355	0.001	-0.119	-0.031
PSWITCH	Direct	AP_EFF	0.085	0.065	1.301	0.193	-0.043	0.213
PSWITCH	Total	AP_EFF	-0.136	0.066	-2.067	0.039	-0.264	-0.007
PSWITCH	Total indirect	AP_EFF	-0.220	0.027	-8.294	0.000	-0.272	-0.168
PSWITCH	Direct	AP_INT	-0.003	0.062	-0.054	0.957	-0.124	0.117
PSWITCH	Total	AP_INT	0.053	0.064	0.830	0.407	-0.072	0.178
PSWITCH	Total indirect	AP_INT	0.056	0.023	2.465	0.014	0.012	0.101
PSWITCH	Direct	RV3	-0.398	0.035	-11.397	0.000	-0.467	-0.330
GEN	Direct	INS_EFF	0.016	0.023	0.695	0.487	-0.029	0.061
GEN	Total	INS_EFF	0.114	0.032	3.549	0.000	0.051	0.178
GEN	Total indirect	INS_EFF	0.099	0.029	3.423	0.001	0.042	0.155
GEN	Direct	AP_EFF	-0.093	0.023	-3.991	0.000	-0.139	-0.047
GEN	Total	AP_EFF	0.196	0.031	6.223	0.000	0.134	0.257
GEN	Total indirect	AP_EFF	0.289	0.029	10.136	0.000	0.233	0.345
GEN	Direct	AP_INT	0.003	0.022	0.148	0.882	-0.040	0.046
GEN	Total	AP_INT	-0.071	0.032	-2.239	0.025	-0.133	-0.009
GEN	Total indirect	AP_INT	-0.074	0.029	-2.561	0.010	-0.131	-0.017
GEN	Direct	RV3	0.522	0.025	21.087	0.000	0.474	0.571
BENEV	Direct	INS_EFF	-0.025	0.017	-1.492	0.136	-0.058	0.008
BENEV	Direct	AP_EFF	0.048	0.018	2.705	0.007	0.013	0.082
BENEV	Direct	AP_INT	-0.002	0.016	-0.109	0.913	-0.032	0.029
BENEV	Direct	RV3	0.062	0.031	2.013	0.044	0.002	0.122
REV	Direct	INS_EFF	0.024	0.035	0.686	0.492	-0.045	0.094
REV	Direct	AP_EFF	0.175	0.039	4.443	0.000	0.098	0.253
REV	Direct	AP_INT	0.037	0.035	1.050	0.294	-0.032	0.106
REV	Direct	RV3	-0.340	0.067	-5.097	0.000	-0.471	-0.209
AP_EFF	RV3	GEN	0.289	0.029	10.136	0.000	0.233	0.345
AP_EFF	RV3	PSWITCH	-0.220	0.027	-8.294	0.000	-0.272	-0.168
AP_INT	RV3	GEN	-0.074	0.029	-2.561	0.010	-0.131	-0.017
AP_INT	RV3	PSWITCH	0.056	0.023	2.465	0.014	0.012	0.101
INS_EFF	RV2	RV3	0.179	0.041	4.332	0.000	0.098	0.259
INS_EFF	RV3	GEN	0.005	0.025	0.216	0.829	-0.043	0.054
INS_EFF	RV3.RV2	GEN	0.093	0.021	4.473	0.000	0.052	0.134
INS_EFF	RV3	PSWITCH	-0.004	0.019	-0.216	0.829	-0.041	0.033
INS_EFF	RV3.RV2	PSWITCH	-0.071	0.017	-4.281	0.000	-0.104	-0.039

Table 10: Main Model Analyses w/ All Participants

outcome	path	pred	est	se	est_se	pval	low2.5	up2.5
RV2	Direct	INS_EFF	0.139	0.038	3.628	0.000	0.064	0.214
ER2	Direct	INS_EFF	-0.118	0.040	-2.972	0.003	-0.197	-0.040
RV3	Direct	RV2	0.748	0.045	16.492	0.000	0.659	0.836
RV3	Direct	ER2	-0.019	0.056	-0.331	0.741	-0.128	0.091
RV3	Direct	INS_EFF	0.006	0.034	0.181	0.857	-0.060	0.072
RV3	Total	INS_EFF	0.112	0.038	2.915	0.004	0.037	0.188
RV3	Total indirect	INS_EFF	0.106	0.028	3.734	0.000	0.050	0.162
RV3	Direct	AP_EFF	0.334	0.039	8.636	0.000	0.258	0.410
RV3	Direct	AP_INT	-0.113	0.038	-2.956	0.003	-0.187	-0.038
ER3	Direct	RV2	-0.054	0.052	-1.041	0.298	-0.157	0.048
ER3	Direct	ER2	0.720	0.051	14.020	0.000	0.619	0.820
ER3	Direct	INS_EFF	0.026	0.038	0.688	0.492	-0.048	0.100
ER3	Total	INS_EFF	-0.067	0.039	-1.708	0.088	-0.144	0.010
ER3	Total indirect	INS_EFF	-0.093	0.029	-3.148	0.002	-0.151	-0.035
ER3	Direct	AP_EFF	-0.239	0.043	-5.629	0.000	-0.323	-0.156
ER3	Direct	AP_INT	-0.004	0.041	-0.107	0.915	-0.084	0.075
PSWITCH	Direct	INS_EFF	-0.020	0.060	-0.338	0.735	-0.139	0.098
PSWITCH	Total	INS_EFF	-0.089	0.064	-1.396	0.163	-0.214	0.036
PSWITCH	Total indirect	INS_EFF	-0.069	0.023	-3.033	0.002	-0.113	-0.024
PSWITCH	Direct	AP_EFF	0.074	0.065	1.124	0.261	-0.055	0.202
PSWITCH	Total	AP_EFF	-0.136	0.066	-2.066	0.039	-0.264	-0.007
PSWITCH	Total indirect	AP_EFF	-0.209	0.027	-7.837	0.000	-0.261	-0.157
PSWITCH	Direct	AP_INT	-0.007	0.061	-0.115	0.908	-0.127	0.113
PSWITCH	Total	AP_INT	0.053	0.064	0.830	0.406	-0.072	0.178
PSWITCH	Total indirect	AP_INT	0.060	0.023	2.580	0.010	0.014	0.106
PSWITCH	Direct	RV3	-0.538	0.069	-7.763	0.000	-0.674	-0.402
PSWITCH	Direct	ER3	0.122	0.083	1.466	0.143	-0.041	0.285
GEN	Direct	INS_EFF	0.025	0.020	1.252	0.211	-0.014	0.065
GEN	Total	INS_EFF	0.114	0.032	3.566	0.000	0.052	0.177
GEN	Total indirect	INS_EFF	0.089	0.028	3.153	0.002	0.034	0.144
GEN	Direct	AP_EFF	-0.087	0.020	-4.230	0.000	-0.127	-0.046
GEN	Total	AP_EFF	0.195	0.031	6.204	0.000	0.133	0.257
GEN	Total indirect	AP_EFF	0.282	0.029	9.805	0.000	0.225	0.338
GEN	Direct	AP_INT	-0.010	0.020	-0.510	0.610	-0.048	0.028
GEN	Total	AP_INT	-0.071	0.031	-2.261	0.024	-0.132	-0.009
GEN	Total indirect	AP_INT	-0.061	0.028	-2.161	0.031	-0.116	-0.006
GEN	Direct	RV3	0.553	0.043	12.927	0.000	0.469	0.637
GEN	Direct	ER3	-0.404	0.045	-8.907	0.000	-0.493	-0.315
BENEV	Direct	INS_EFF	-0.027	0.017	-1.537	0.124	-0.061	0.007
BENEV	Direct	AP_EFF	0.062	0.018	3.379	0.001	0.026	0.098
BENEV	Direct	AP_INT	0.004	0.016	0.232	0.817	-0.028	0.036
BENEV	Direct	RV3	0.068	0.043	1.578	0.115	-0.017	0.153
BENEV	Direct	ER3	0.076	0.038	1.998	0.046	0.001	0.151
REV	Direct	INS_EFF	0.022	0.035	0.634	0.526	-0.047	0.092
REV	Direct	AP_EFF	0.154	0.040	3.810	0.000	0.075	0.233
REV	Direct	AP_INT	0.023	0.036	0.637	0.524	-0.047	0.092

Table 10: Main Model Analyses w/ All Participants *(continued)*

outcome	path	pred	est	se	est_se	pval	low2.5	up2.5
REV	Direct	RV3	-0.479	0.091	-5.270	0.000	-0.657	-0.301
REV	Direct	ER3	-0.075	0.081	-0.935	0.350	-0.233	0.083
AP_EFF	ER3	GEN	0.097	0.020	4.838	0.000	0.058	0.136
AP_EFF	RV3	GEN	0.185	0.024	7.624	0.000	0.137	0.232
AP_EFF	ER3	PSWITCH	-0.029	0.021	-1.384	0.166	-0.071	0.012
AP_EFF	RV3	PSWITCH	-0.180	0.030	-5.916	0.000	-0.239	-0.120
AP_INT	ER3	GEN	0.002	0.016	0.107	0.915	-0.030	0.034
AP_INT	RV3	GEN	-0.062	0.021	-2.976	0.003	-0.103	-0.021
AP_INT	ER3	PSWITCH	-0.001	0.006	-0.091	0.927	-0.012	0.011
AP_INT	RV3	PSWITCH	0.061	0.022	2.765	0.006	0.018	0.104
INS_EFF	ER2	RV3	0.002	0.007	0.297	0.767	-0.012	0.017
INS_EFF	RV2	RV3	0.104	0.029	3.527	0.000	0.046	0.162
INS_EFF	ER2	ER3	-0.085	0.030	-2.860	0.004	-0.144	-0.027
INS_EFF	RV2	ER3	-0.008	0.008	-0.938	0.348	-0.023	0.008
INS_EFF	ER3	GEN	-0.010	0.015	-0.687	0.492	-0.040	0.019
INS_EFF	ER3.ER2	GEN	0.034	0.013	2.731	0.006	0.010	0.059
INS_EFF	ER3.RV2	GEN	0.003	0.003	0.930	0.352	-0.003	0.009
INS_EFF	RV3	GEN	0.003	0.019	0.179	0.858	-0.033	0.040
INS_EFF	RV3.ER2	GEN	0.001	0.004	0.287	0.774	-0.007	0.010
INS_EFF	RV3.RV2	GEN	0.057	0.016	3.486	0.000	0.025	0.090
INS_EFF	ER3	PSWITCH	0.003	0.006	0.561	0.575	-0.008	0.014
INS_EFF	ER3.ER2	PSWITCH	-0.010	0.008	-1.262	0.207	-0.027	0.006
INS_EFF	ER3.RV2	PSWITCH	-0.001	0.001	-0.706	0.480	-0.003	0.002
INS_EFF	RV3	PSWITCH	-0.003	0.018	-0.178	0.859	-0.039	0.033
INS_EFF	RV3.ER2	PSWITCH	-0.001	0.004	-0.291	0.771	-0.009	0.007
INS_EFF	RV3.RV2	PSWITCH	-0.056	0.017	-3.242	0.001	-0.090	-0.022

Analysis of Essay Ratings

In this experiment, we collected participants' ratings of the other group members' essays. Every participant rated three essays, each using six items. Across all essays and all items, correlations were positive and ranged from moderate (min r = .204) to high (max r = .674), and a single factor model for all items exhibited high internal consistency, Cronbach's $\alpha = .91$. We created a composite rating for each participant that represented their average rating across all items and essays. Participants' own ratings may have impacted how they responded to the insult (e.g., people who provide negative feedback may not be as impacted by negative feedback), which in turn could impact their self-reported perceptions of relationship value and forgiveness. Because participants did not know who authored each essay at the time it was evaluated, and because this occurred prior to any transgression, these ratings should not systematically differ by condition. Indeed, regressing average essay ratings with manipulated relationship value, apology, and their interaction revealed no significant effects (see Table 11).

We correlated average essay ratings with post-transgression relationship value, post-apology relationship value, and self-reported forgiveness. These analyses revealed no correlation between participants' average essay ratings of other participants' essays and their post-transgression relationship value, $t(968) = 1.328$, $p = .185$, $r = .043$, 95% CI [-.020, .105]. There was, however, a statistically significant correlation between participants' average ratings of other participants' essays and post-apology relationship value, $t(968) = 3.062$, $p = .002$, $r = .098$, 95% CI [.035, .160], as well as between essay ratings and self-reported forgiveness, $t(963) = 4.955$, $p < .001$, $r = .158$, 95% CI [.096, .219]. This indicates that people who evaluated other participants' essays generously tended also be evaluate their insulters as having relatively high relationship value and forgivability. These results may suggest that participants' ratings of others' essays are an indicator of individual differences in agreeableness.

Test for Systematic Variance in Essay Ratings Across Conditions

Table 11: Effect of Condition Assignment on Participants' Average Essay Ratings

Effect	Estimate	Std. Error	t value	Pr(>|t|)
(Intercept)	6.9647755	0.0389774	178.6876390	0.0000000
Transgressor Value	-0.0104798	0.0389774	-0.2688680	0.7880886
Apology	-0.0235441	0.0389774	-0.6040457	0.5459549
Transgressor Value:Apology	-0.0687067	0.0389774	-1.7627340	0.0782612

Correlating Essay Ratings with Outcomes

Table 12: Average Essay Ratings Correlated with Relationship Value and Forgiveness

outcome	estimate	t(df)	p.value	LB	UB
Post-Transgression RV	0.0426336	1.328 (968)	0.1846052	-0.0203659	0.1052959
Post-Apology RV	0.0979316	3.062 (968)	0.0022621	0.0352038	0.1598909
Self-Report Forgiveness	0.1576873	4.955 (963)	0.0000009	0.0955302	0.2186196

Appendix A. Relationship Closeness Induction Task for Mturk Samples

Section 1

"You have 1 minute and 30 seconds to discuss these questions:"

1. What is your first name?
2. How old are you?
3. Where are you from?
4. How long have you been on MTurk?
5. What was your favorite HIT on MTurk? Why?

Section 2

"You have three minutes to discuss these questions:"

1. What are your hobbies?
2. What would be the perfect lifestyle for you?
3. What is something you have always wanted to do but probably never will be able to do?
4. If you could travel anywhere in the world, where would you go and why?
5. What is one strange thing that has happened to you on the internet?
6. What is one embarrassing thing that has happened to you on the internet?
7. What is one thing happening in your life that makes you stressed out?
8. If you could change anything that happened to you in high school, what would that be?
9. If you could change one thing about yourself, what would that be?
10. Do you miss your family?
11. What is one habit you'd like to break?

Section 3

"You have five minutes to discuss these questions:"

1. If you could have one wish granted, what would that be?
2. Is it difficult or easy for you to meet people? Why?
3. Describe the last time you felt lonely.
4. What is one emotional experience you've had with a good friend?
5. What is one of your biggest fears?
6. What is your most frightening early memory?
7. What is your happiest early childhood memory?
8. What is one thing about yourself that most people would consider surprising?
9. What is one recent accomplishment that you are proud of?
10. Tell me one thing about yourself that most people who already know you don't know?

Appendix B. Items for Providing Feedback on Essays

Using the scales below, please give your honest opinion about the essay you just read.

Please indicate how [low-anchor] or [high-anchor] you felt the [first/second/third] essay was.

1. 1 = unintelligent; 9 = intelligent
2. 1 = boring; 9 = thought-provoking
3. 1 = unfriendly; 9 = friendly
4. 1 = illogical; 9 = logical
5. 1 = unrespectable; 9 = respectable
6. 1 = irrational; 9 = rational

Please write a 1-2 sentence review of the [first/second/third] essay. We are just interested in your general reactions (e.g., was it well-written? was the logic sound?).

Appendix C. Relationship Value and Exploitation Risk scale for Non-Close Others (RVEX-NCO)

Even though you will never meet [Target], we are interested in how you think you would feel if you were to encounter him/her in your daily life.

Please use the scale below to indicate how much each statement below reflects how you think you would feel about interacting with [Target] in the context of your everyday life. 1 = strongly disagree 2 = mildly disagree 3 = agree and disagree equally 4 = mildly agree 5 = strongly agree 6 = I prefer not to answer

1. I feel that he/she could become an important person in my life. (Relationship Value)
2. I might find him/her to be a valuable friend or acquaintance. (Relationship Value)
3. I think we would discover common interests. (Relationship Value)
4. I do not think a relationship with him/her would be rewarding for me. (Relationship Value)
5. Becoming his/her friend would hold no value for me. (Relationship Value)
6. I think I could develop a meaningful relationship with him/her. (Relationship Value)
7. I would worry that he/she would take advantage of me. (Exploitation Risk)
8. He/she would surely try to exploit me for his/her own gains. (Exploitation Risk)
9. He/she would eventually hurt or offend me in some way. (Exploitation Risk)
10. I would be suspicious about how he/she might treat me. (Exploitation Risk)
11. I would be worried about interacting with him/her. (Exploitation Risk)
12. I would not be able to depend on him/her. (Exploitation Risk)

Appendix D. Transgression Related Interpsonal Motivations Inventory for Non-Close Others (TRIM-NCO)

For the following questions, please indicate your current thoughts and feelings about [Target] using the scale below. Even though you will never encounter [Target] again, we are interested in how you think you would respond if you were to encounter in your daily life.

1 = strongly disagree 2 = mildly disagree 3 = agree and disagree equally 4 = mildly agree 5 = strongly agree 6 = I prefer not to answer

1. I would want to make him/her pay for treating me badly today.
2. I would try to keep as much distance between the two of us as possible.
3. I would have good will for him/her.
4. I would hope for something bad to happen to him/her.
5. I would have nothing to do with him/her.
6. I would try to put aside any reservations I had in order to develop a good relationship with him/her.
7. I would not trust him/her.
8. I would be willing to work toward a positive relationship with him/her.
9. I would act warmly towards him/her.
10. I would avoid contact with him/her.
11. I would be very happy to interact with him/her.
12. I would want to get even with him/her.
13. I would try to give up negative feelings toward him/her.
14. I would avoid working with him/her.
15. I would be willing to let go of my anger towards him/her.
16. I would want to seek revenge.
17. I would try to avoid him/her.
